# Determination of Thiol Protonation States by Sulfur X-ray Spectroscopy in Biological Systems

**DOI:** 10.1021/acs.jpclett.4c03247

**Published:** 2025-02-27

**Authors:** Ryan D. Ribson, Alec H. Follmer, Jeffrey T. Babicz, Victor Sosa Alfaro, Ryan G. Hadt, Mark S. Hunter, Mark A. Wilson, Dimosthenis Sokaras, Roberto Alonso-Mori

**Affiliations:** † Linac Coherent Light Source, SLAC National Accelerator Laboratory, Menlo Park, California 94025, United States; ‡ Department of Chemistry, University of California, Davis, One Shields Avenue, Davis, California 95616, United States; § SSRL, SLAC National Accelerator Laboratory, Menlo Park, California 94025, United States; ∥ Division of Chemistry and Chemical Engineering, Arthur Amos Noyes Laboratory of Chemical Physics, California Institute of Technology, Pasadena, California 91125, United States; ⊥ Department of Biochemistry, University of Nebraska, Lincoln, Nebraska 68588, United States

## Abstract

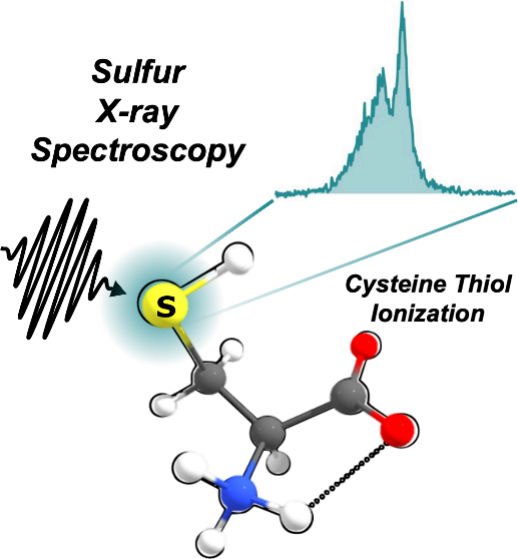

Cysteine is one of the most functionally diverse of the proteinogenic amino acids, owing to its reactive thiol side chain that can undergo deprotonation to form a strongly nucleophilic thiolate. However, few techniques can directly interrogate sulfur charge and covalency in cysteine, particularly in proteins. X-ray spectroscopies provide an element specific probe of sulfur. We demonstrate the sensitivity of S Kβ and Kα X-ray emission spectroscopy (XES) to cysteine ionization and compare it to S K-edge X-ray absorption spectroscopy (XAS) in the physiologically relevant biomolecules l-cysteine and *N*-acetyl-l-cysteine at room temperature in solution phase. Kβ XES and K-edge XAS are most sensitive to chemical changes at the cysteine thiol and can be used to evaluate the composition of thiol/thiolate mixtures. These results provide a foundation for assessing the p*K*_a_ of functionally significant cysteine residues in proteins and open the door to time-resolved studies of cysteine-dependent enzymes.

Sulfur is a vital nutrient for all living organisms, serving in a broad range of biological functions from cell signaling and metabolism to protein structure and function. In proteins, sulfur is incorporated as the defining component of the side chains of two amino acids: cysteine (Cys) and methionine (Met). Cys is particularly important in this context due to the versatility of its thiol (-SH) group, which allows it to function as a potent nucleophile in enzymatic reactions as well as form disulfide bonds (S–S) with other Cys residues to stabilize protein structures.^1−3^ Moreover, its electron-rich side chain enables Cys residues to participate in redox chemistry and metal coordination, making it central to a number of important biological processes that require the shuttling of protons and electrons.^4^

The diverse functionality of Cys is largely attributable to its ability to form a reactive thiolate anion (-S^–^) upon deprotonation of its thiol side chain. The ionization from thiol to thiolate is governed by its p*K*_a_, which is ∼8.3 for free Cys in solution–slightly above the physiological range.^5^ However, the microenvironments created within enzyme active sites can lower the p*K*_a_ of Cys residues as low as ∼3.3 to promote thiolate formation at physiological pH, enabling the chemistry of important enzymes such as Cys proteases, hydratases, thiol–disulfide oxidoreductases, and many others.^6−12^ Tuning the electronics of Cys is also crucial for the function of metalloenzymes, like cytochromes P450, and electron-transfer proteins, such as cupredoxins and ferredoxins. In these metalloproteins, the degree of electron localization on the sulfur atom affects the covalency of the metal–sulfur bond, which, in turn, influences thermodynamic properties like the redox potential of the metal center.^4,13−17^

Determining the p*K*_a_ and electronic structure of Cys residues is critical for understanding their roles in protein function and linking p*K*_a_ measurements to complementary structural information is essential for understanding how a protein fold controls the electronic properties of cysteine. However, few techniques are available that can selectively probe the electronic states of sulfur. For example, while Cys thiol deprotonation can be measured by UV–vis spectroscopy, the thiolate’s absorption maximum around 240 nm is frequently masked by the strong absorbance of the protein backbone in the 220–250 nm range and overlapping contributions from aromatic residues in the 260–280 nm range.^18,19^ Other spectroscopic approaches, such as NMR can be complex and indirect, often requiring multiple control experiments as sulfur lacks high natural abundance NMR-sensitive isotopes.^20,21^ Furthermore, Cys-mediated redox events, including electron and proton transfer and the formation of transition state intermediates occur on milli- to nanosecond time scales, precluding their analysis by most standard time-resolved techniques.

X-ray spectroscopies provide an element specific probe of S chemistry and are made possible at synchrotron and X-ray free electron laser (XFEL) facilities.^22−24^ S K-edge X-ray absorption spectroscopy (XAS) reports on transitions from the S 1s orbital to unoccupied orbitals with S *n*p character. Overlapping in the X-ray absorption near edge structure (XANES) of the XAS spectrum is the K-edge ionization of the 1s electron into unbound continuum states and potential multiple scattering effects. In S X-ray emission spectroscopy (XES), the Kα line results from transitions involving a 2p electron filling the 1s core hole, whereas the Kβ line involves transitions from orbitals with 3p character to the 1s hole (Figure 1). The 3p orbitals are the valence orbitals of S and as a result, the Kβ mainline provides a direct probe of the S valence shell (valence-to-core transitions). While XAS typically requires a tunable monochromatic X-ray source, XES provides the advantage that spectra can be collected under nonresonant or resonant conditions. When the emission line is collected while scanning the incident X-ray energy through an edge absorption, this is known as resonant inelastic X-ray scattering (RIXS), which produces a 2D data set along both incident and emitted X-ray energy axes.^22−24^

**Figure 1:**
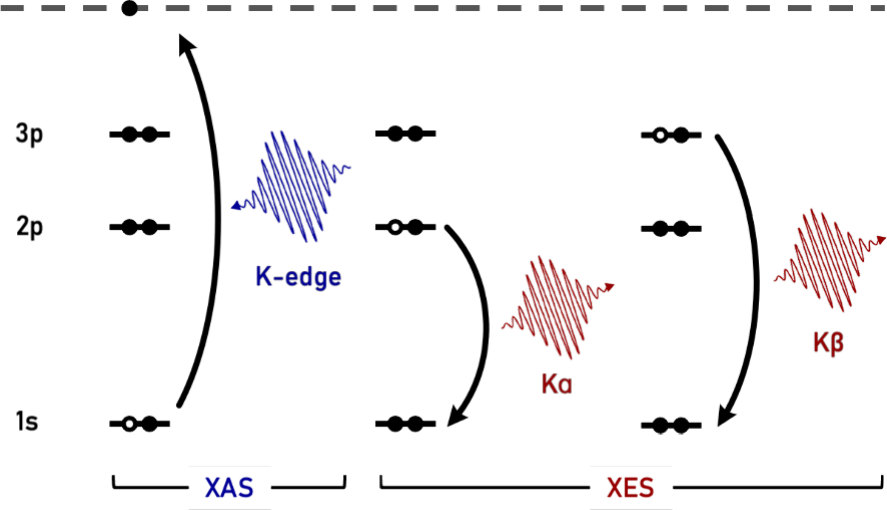
General scheme showing the orbital contributions to K-edge XAS in which a 1s electron is ionized to the continuum, Kα XES in which a 2p electron fills the 1s core hole, and Kβ XES in which a 3p electron fills the 1s core hole.

S K-edge XAS and K line XES fall within the tender X-ray region (2–5 keV). Previous reports have demonstrated the sensitivity of S K-edge XAS to the chemical environment of sulfur in biologically relevant molecules, including Cys.^25−31^ Most of these studies were conducted on frozen solutions and not under physiologically relevant conditions. While there are fewer studies detailing such effects via S K line XES, the S Kβ line has shown comparable chemical sensitivity to XAS.^32−35^ Although these techniques can provide complementary information, XES provides distinct advantages when considering the extension of these experiments to time-resolved studies on nonequilibrium phenomena in sulfur containing samples at XFELs and some advanced synchrotron beamlines. XFELs deliver trains of high intensity X-ray pulses with tunable X-ray energies and the use of energy dispersive detectors allows for the collection of a full XES spectrum on a shot-by-shot basis.^36,37^ Time-resolved nonresonant XES thus only requires scanning the time axis to generate a complete data set. In contrast, time-resolved XAS requires scanning of the incident X-ray energy in addition to scanning the time axis, extending the time required to collect full 2D data sets with reasonable statistics. The dispersive mode XES collection at a single incident energy also facilitates simultaneous collection of X-ray forward scattering data (like solution scattering or diffraction) allowing for full integration with existing serial crystallography configurations at synchrotrons and XFELs.

Toward the development of XES techniques to assess Cys reactivity in protein chemistry, we study here the pH-dependent thiol–thiolate interconversion of N-acetyl-l-cysteine (NAC) and Cys in aqueous solution at room temperature by S K line XES and compare its sensitivity to the complementary K-edge XAS techniques. Compared to free Cys, NAC better resembles Cys upon incorporation into the protein backbone due to the acetyl group capping the amine moiety. The N-acetyl cap also causes NAC and Cys to display different pH-dependent behavior as the amine group in Cys has a similar microscopic p*K*_a_ as the thiol, resulting in a complex competitive equilibrium (Figure 2). This complex coupled ionization allows us to test the ability of XES techniques to distinguish subtle changes in the ionization equilibria in the system.

**Figure 2:**
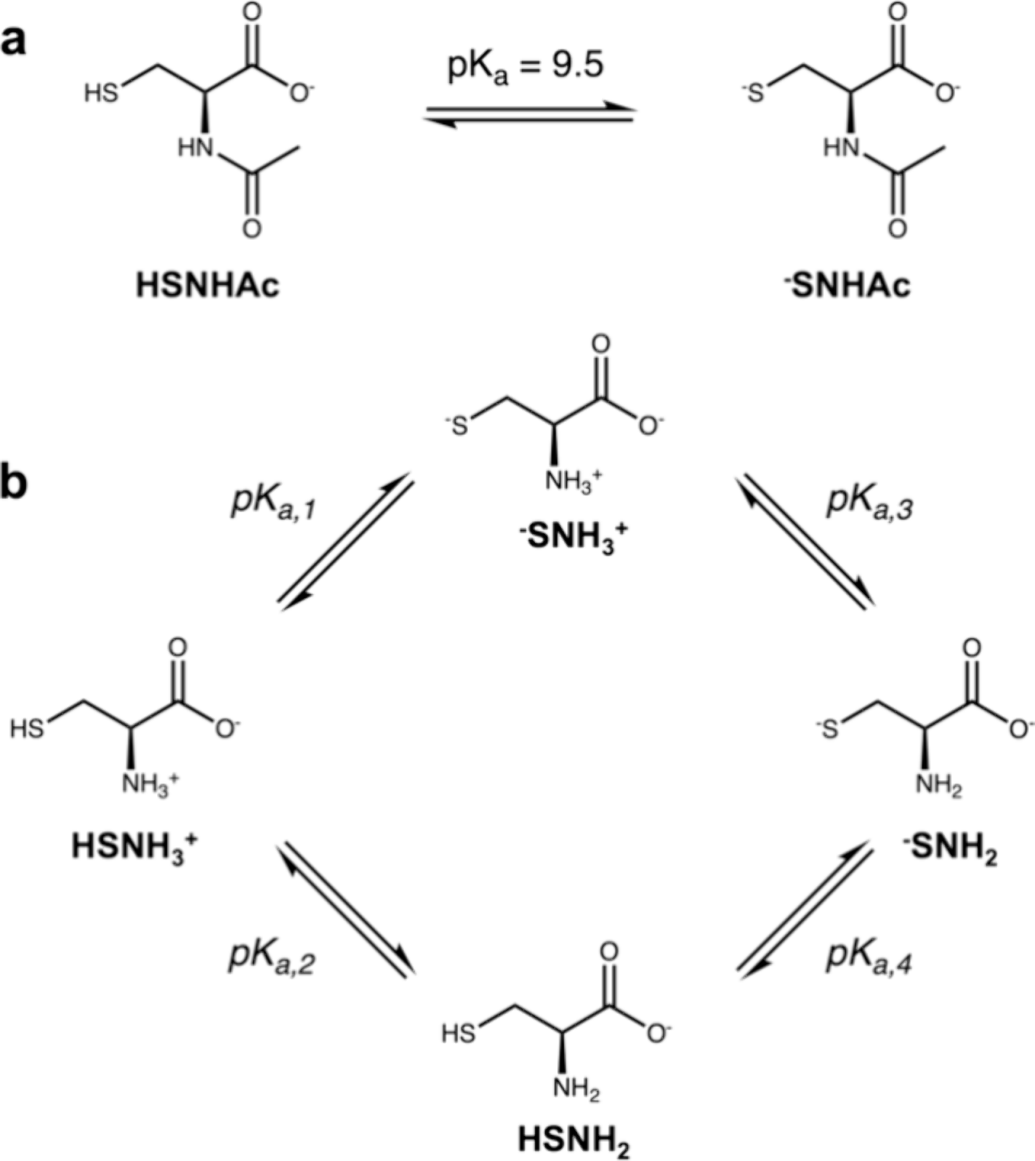
(a) The p*K*_a_ of thiol (**HSNHAc**)/thiolate (^**–**^**SNHAc**) interconversion in NAC is 9.5. (b) Cys has four relevant protonation states in the pH range from 6 to 13: **HSNH**_**3**_^**+**^, **HSNH**_**2**_, ^**–**^**SNH**_**3**_^**+**^, and ^**–**^**SNH**_**2**_; the reported microscopic p*K*_a_’s describing their interconversion are p*K*_a,1_ = 8.53, p*K*_a,2_ = 8.86, p*K*_a,3_ = 10.03, and p*K*_a,4_ = 10.36.^18^

We begin our investigation by focusing on NAC. NAC is a well-established model for peptide-incorporated Cys and is also a small molecule therapeutic.^38−40^ We collected nonresonant Kβ XES, nonresonant Kα XES, and Kα RIXS on 50 mM solutions of NAC at pH values of 6.2, 8.5, 9.5, 10.4, and 13, a range expected to cover the ionization of the thiol. The thiol/thiolate equilibrium of NAC has a reported p*K*_a_ of 9.5 (Figure 2a) and is well described by the Henderson–Hasselbalch equation.^39^ At pH 6, we expect nearly 100% of the NAC molecules in solution to be in the thiol form **HSNHAc**; at pH 13, we expect 100% of the NAC molecules to be thiolate ^**–**^**SNHAc**; and at pH 9.5, we expect a 50/50 mixture of the two species. As a result, we take the pH 6.2 and 13 data to represent the pure thiol and thiolate, respectively.

We observe a significant change in the shape of the Kβ spectrum moving from pH 6.2 (thiol) to pH 13 (thiolate) (Figure 3a). Performing singular value decomposition (SVD) on the data matrix of spectra by pH reveals two significant components contributing to the data (Figure S6). This observation is consistent with the expectation that two chemical species - **HSNHAc** and ^**–**^**SNHAc** - are spectrally distinguishable and contribute to the pH-dependent changes observed in the data.

**Figure 3:**
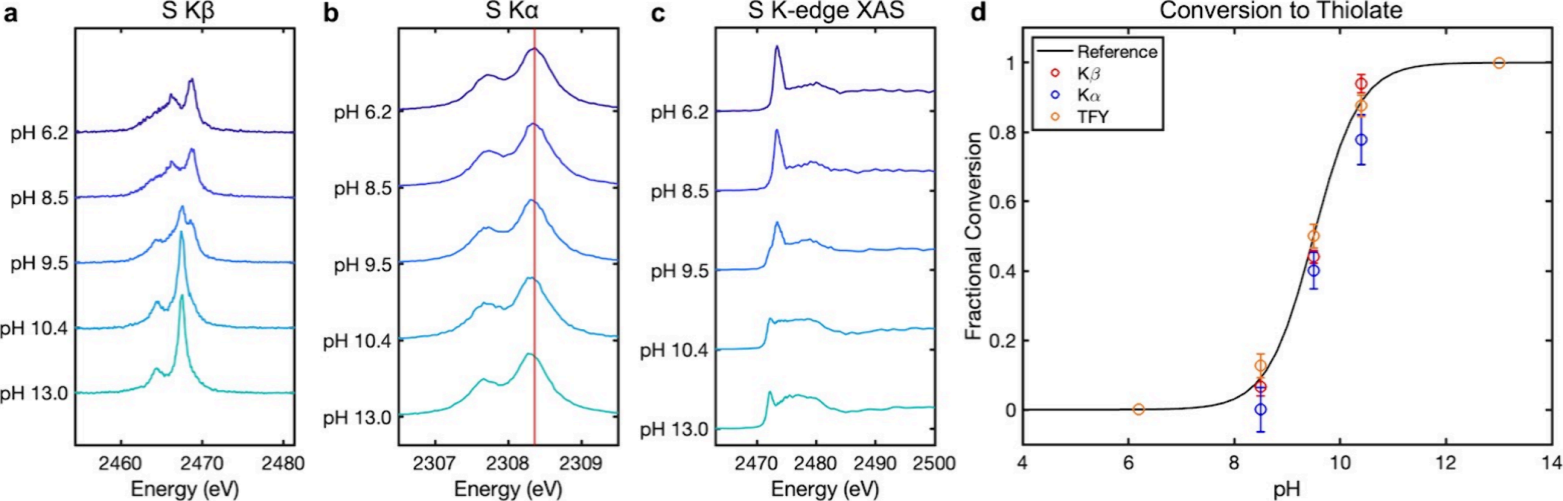
(a) Nonresonant S Kβ XES spectra of NAC collected at five pH points between pH 6 and 13, showing significant spectral changes as the equilibrium shifts from thiol to thiolate. (b) Nonresonant S Kα XES spectra of NAC collected at the same pH points; the red line reflects the energy position of the fitted Kα_1_ line at pH 6.2. (c) S K-edge TFY XAS spectra of NAC collected at the same pH points. (d) Fractional conversion to thiolate calculated from fitting intermediate pH points to combinations of “pure” thiol and thiolate basis spectra. The results from the Kβ, Kα, and TFY fits are overlaid against a reference curve reflecting the fractional conversion of a system with p*K*_a_ = 9.5 using the Henderson–Hasselbalch equation. Error bars represent the 95% confidence interval from the least-squares fit.

We compare the pH 6.2 and pH 13 NAC S Kβ spectra to the simulated valence-to-core (VtC) XES obtained for **HSNHAc** and ^**–**^**SNHAc** DFT optimized structures, respectively, and find good agreement between the line-broadened theoretical predictions and the experimental results (Figure 4). The orbital contributions to the most intense transitions in the DFT spectra are summarized in the SI (Tables S4 and S7). For **HSNHAc**, the dominant contributions to the high energy feature are transitions from S nonbonding 3p orbitals to the S 1s orbital, whereas the broad bands on the low energy side of the spectrum arise due to transitions from hybrid S–H and S–C σ-bonding orbitals with S 3p character. In the case of ^**–**^**SNHAc**, the intense high energy feature is similarly due to transitions involving nonbonding S 3p orbitals and the lower energy band involves the S–C σ-bonding orbital. As ^**–**^**SNHAc** has one fewer covalent bond to S than **HSNHAc**, the low energy portion of the thiolate spectrum is much sharper than the broadness observed for the thiol due to the fewer hybrid bonding orbitals with major S 3p character.

**Figure 4:**
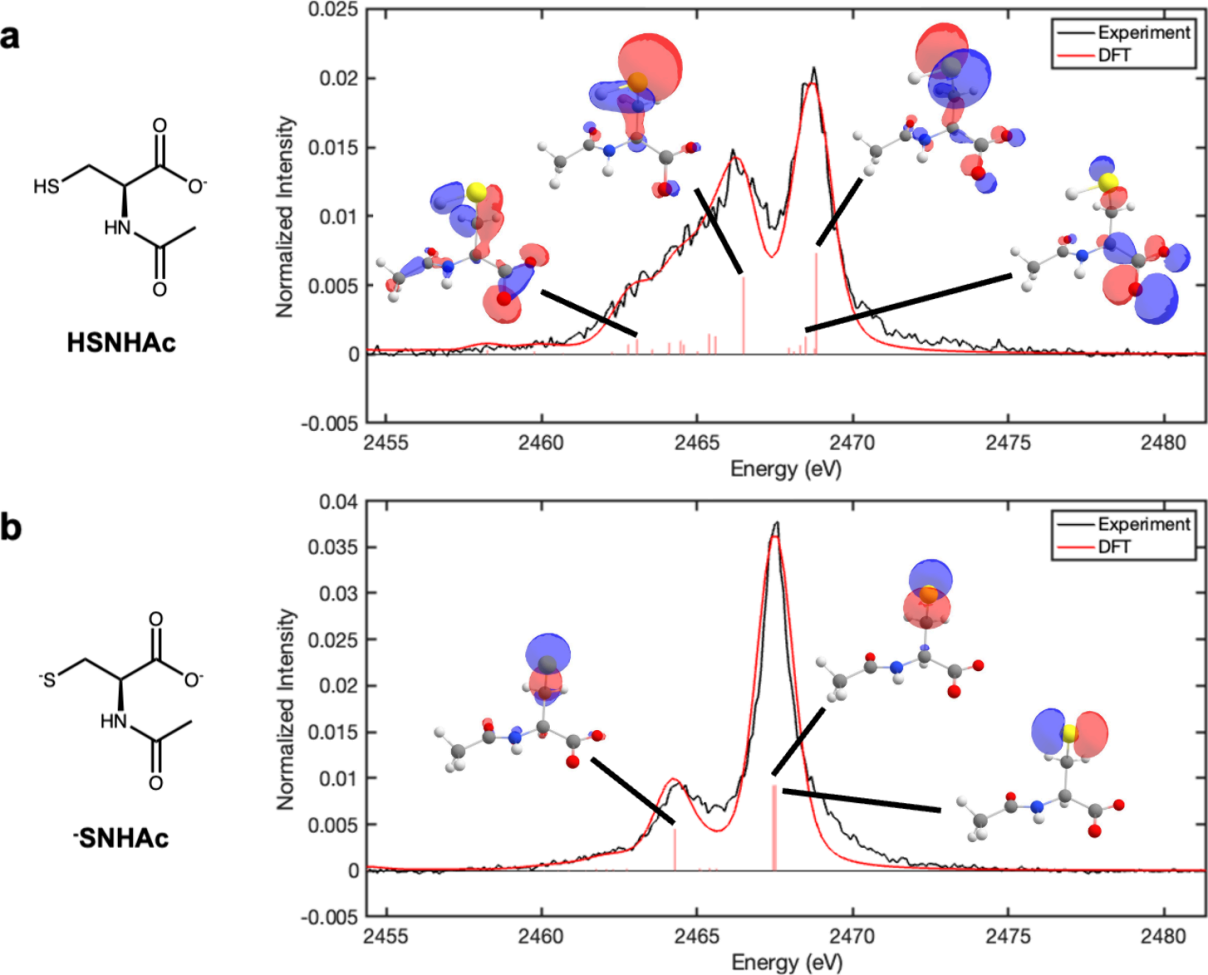
S Kβ XES of NAC at (a) pH 6.2 and (b) pH 13 compared to the DFT simulated Kβ spectra of **HSNHAc** and ^**–**^**SNHAc**, respectively. Orbitals shown are the initial orbitals involved in transitions to the 1s core hole that give rise to a select number of the dominant features in the spectra.

The change in the S Kα mainline emission of NAC (Figure 3b) going from thiol to thiolate is much more subtle than the change observed for the Kβ but consists of a shift in the energy of the Kα_1_ and Kα_2_ peaks from higher (thiol) to lower (thiolate) energies. SVD of the Kα data matrix also identifies two independent components (Figure S12). We fit the pH 6.2 and pH 13 Kα spectra to a sum of two Lorentzian functions representing the Kα_1_ and Kα_2_ lines (Figure S10) with the Lorentzian line width fixed to the S 1s core hole lifetime broadening (0.522 eV).^41^ The optimized centroid positions are then taken as the Kα_1_/Kα_2_ energies (Table S2). The thiol (thiolate) Kα_1_ centroid is fitted to 2308.374 ± 0.002 eV (2308.326 ± 0.002 eV) and the Kα_2_ centroid fitted to 2307.710 ± 0.004 eV (2307.661 ± 0.004 eV). Thus, the Kα_1_/Kα_2_ energy shifts by ∼0.05 eV to lower energies going from thiol to thiolate.

As the S Kα energy is largely dictated by charge screening effects, it is sensitive to changes in valence orbital population. Indeed, it has been observed that the S Kα generally trends from higher energy to lower energy across the series of S oxidations states 6+ to 2-. This trend similarly holds by relating the S Kα energy to computed Mulliken charge densities of the s and p orbitals of S.^42^ A greater population of the valence 3p orbitals (more reduced) leads to a greater degree of screening of the nuclear charge (lower Z_eff_), which approximately lowers the energy gap between the 1s and 2p orbitals (lower Kα energy).

Although **HSNHAc** and ^**–**^**SNHAc** both exhibit sulfur in the formal 2- oxidation state, the DFT-computed Mulliken reduced population analysis shows an increase in the electron density in S p orbitals going from thiol to thiolate (Table S10). This result makes sense as the electrons that are covalently shared in the S–H bond in the thiol become localized in S 3p orbitals in the thiolate. The thiolate thus resembles a more reduced S, leading to the lower energy Kα emission, albeit with a much smaller shift than might be expected for a formal change in oxidation state.

In addition to the nonresonant emission experiments, we collected Kα RIXS scans for each pH point (Figures S16–S20). Integrating the emission energy axis produces a total fluorescence yield (TFY) S K-edge XAS spectrum. Observing the change in the TFY XAS spectra as a function of pH, we find the white line of the spectrum shifts from higher to lower energy going from thiol to thiolate (Figure 3c). The two protonation states are thus well differentiated by XAS, similar to the Kβ data. Time-dependent density functional theory (TDDFT) was used to simulate transitions from the 1s orbital to unoccupied valence and higher-lying orbitals for **HSNHAc** and ^**–**^**SNHAc**. The simulated spectra reasonably agree with the near edge features in the pH 6.2 and 13 data (Figures S50 and S53). The white line in both cases is predominantly attributed to transitions arising from S 1s to S–C or S–H σ* orbitals with some admixture of carboxylate and acetyl π* orbitals.

As previously mentioned, the pH 6.2 and 13 samples are taken to represent pure **HSNHAc** and ^**–**^**SNHAc** speciation, respectively, and the three intermediate pH points (8.5, 9.5, and 10.4) represent mixtures of varying percentages of thiol and thiolate. As a result, the data collected at pH 6.2 and 13 can be used as basis spectra for the thiol and thiolate species, respectively. We fit our intermediate spectra to a linear combination of these basis spectra in order to back out a fractional conversion from thiol to thiolate for comparison to the expected reference curve derived from the Henderson–Hasselbalch equation for a p*K*_a_ of 9.5 (Figure 3d).

The fitted fractional conversions from the Kβ and TFY XAS spectra reasonably reflect the expected values. Although the fitted results from the Kα data follow the expected trend, the absolute fractional conversions deviate more significantly from the reference curve than the Kβ or TFY results. Additionally, the standard errors associated with the Kα fits are in general larger than those for the Kβ and XAS fits. Although we can differentiate between thiol and thiolate in the Kα data, the small magnitude of the shift is likely bordering on what we can resolve with our instrumentation, which we believe contributes to the greater degree of uncertainty.

We extend our approach to Cys by collecting nonresonant Kβ XES, nonresonant Kα XES, and Kα RIXS at pH points 6.4, 8.1, 9.1, 10.9, and 13. Cys exhibits three distinct macroscopic p*K*_a_’s at 1.71, 8.33, and 10.78 reflecting the overall change in ionization state of the molecule.^5^ The first of these reflects the carboxylic acid/carboxylate equilibrium and bears no role in our current analysis. Although the 8.33 and 10.78 p*K*_a_ values are often attributed to ionization of the thiol and amine, respectively, these equilibrium values result from competitive protonation/deprotonation events at both the thiol and amine in this pH range and thus cannot be uniquely assigned to a specific moiety.^18^ This gives rise to pH-dependent behavior that deviates from the traditional Henderson–Hasselbalch equation where it is assumed that we are either treating a monoprotic acid or a polyprotic acid where the K_a_’s of the different protonation events are sufficiently different in magnitude such that we can treat each ionization as a sequential event. Because of this deviation, we must treat the four equilibria shown in Figure 2b and consider the four possible protonation states labeled **HSNH**_**3**_^**+**^, ^**–**^**SNH**_**3**_^**+**^, **HSNH**_**2**_, and ^**–**^**SNH**_**2**_. A derivation of the fractional conversion of thiol to thiolate using the four microscopic p*K*_a_’s can be found in the SI. With the previously reported microscopic p*K*_a_’s from Benesch and Benesch, we can use this equation as a reference for Cys thiolate conversion as a function of pH and note that we also reproduce this curve in our buffer system via UV–vis titration experiments (Figure S26).^18^

The S Kβ spectra of Cys between pH 6.4 and 13 is shown in Figure 5a. As with NAC, the S Kβ spectrum of Cys changes significantly moving from low to high pH. The pH 6.4 spectrum of Cys and the pH 6.2 spectrum of NAC are overlaid in Figure S47a to compare the thiol spectra for both compounds and we find excellent agreement between the two spectra. We observe similar agreement between the Cys and NAC thiolate spectra collected at pH 13 (Figure S47b), suggesting that acetylation of the amine does not significantly affect the spectroscopic signatures of thiol and thiolate observed.

**Figure 5:**
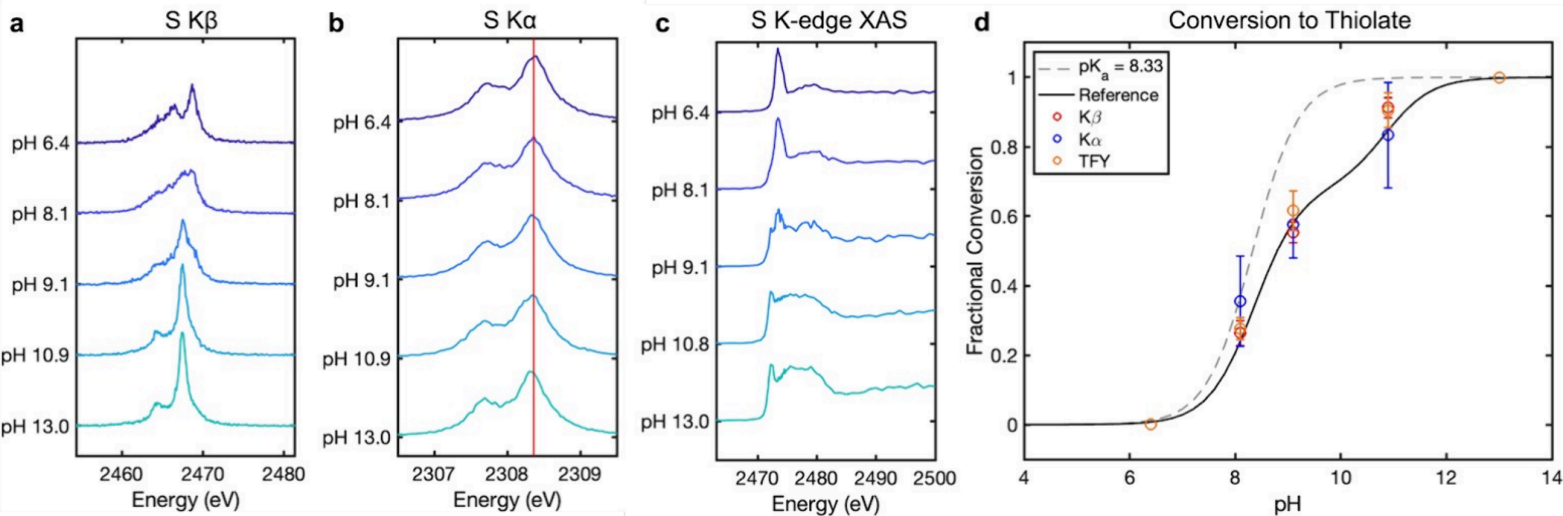
(a) Nonresonant S Kβ XES spectra of Cys collected at five pH points between pH 6 and 13, showing significant spectral changes as the equilibrium shifts from thiol to thiolate. (b) Nonresonant S Kα XES of Cys collected at the same pH points; the red line reflects the energy position of the fitted Kα_1_ line at pH 6.4. (c) S K-edge TFY XAS spectra of Cys collected at the same pH points. (d) Fractional conversion to thiolate calculated from fitting intermediate pH points to combinations of “pure” thiol and thiolate basis spectra. The results from the Kβ, Kα, and TFY fits are overlaid against a reference curve reflecting the expected fractional conversion calculated using the reported microscopic p*K*_a_’s (black line). The Henderson–Hasselbalch curve for a p*K*_a_ of 8.33 is also shown for comparison (gray dashed line). Error bars represent the 95% confidence interval from the least-squares fit.

DFT calculations were carried out for **HSNH**_**3**_^**+**^, ^**–**^**SNH**_**3**_^**+**^, **HSNH**_**2**_, and ^**–**^**SNH**_**2**_. Given the equilibrium outlined in Figure 2, we expect only **HSNH**_**3**_^**+**^ in solution at pH 6.4 and only ^**–**^**SNH**_**2**_ at pH 13. The DFT-predicted S Kβ XES for **HSNH**_**3**_^**+**^ and ^**–**^**SNH**_**2**_ are in good agreement with the experimentally observed spectra at pH 6.4 and 13, respectively (Figures S55 and S58). Analysis of the transitions contributing to the XES spectra of Cys is very similar to that of NAC: the high energy peak is a result of transitions from the S 3p nonbonding electrons to the 1s core hole, whereas the low energy features involve hybrid S–C (and S–H in the case of the **HSNH**_**3**_^**+**^) σ-bonding orbitals.

The Cys S Kα spectrum also exhibits a shift to lower energies going from the thiol at pH 6.4 to the thiolate at pH 13 (Figure 5b). Fitting the spectra at these pH points to a sum of two Lorentzian functions provides an estimate of the Kα_1_/Kα_2_ energies (Table S3) and reveals a shift of ∼0.04 eV, similar to that observed in NAC (0.05 eV). We similarly integrated the S Kα RIXS maps to obtain TFY S K-edge XAS spectra of Cys at each pH point (Figure 5c). The trend in the spectral shape of the K-edge absorption is consistent with what we observed in NAC and is also highly sensitive to thiol vs thiolate composition.

It is reasonable to consider whether our spectroscopy can distinguish these four species **HSNH**_**3**_^**+**^, ^**–**^**SNH**_**3**_^**+**^, **HSNH**_**2**_, and ^**–**^**SNH**_**2**_. By comparing the VtC DFT calculations of the four states (Figure S66), we find that the spectra of the thiolate species (^**–**^**SNH**_**3**_^**+**^ and ^**–**^**SNH**_**2**_) overlap remarkably well in the Kβ region with minimal differences. The thiol species (**HSNH**_**3**_^**+**^ and **HSNH**_**2**_) exhibit largely similar predicted Kβ spectra with subtle differences in the lower energy features. Thus, the DFT leads us to expect that thiol and thiolate species should be distinguished from each other by XES, but that only minor differences may appear within the thiol (**HSNH**_**3**_^**+**^ and **HSNH**_**2**_) and thiolate (^**–**^**SNH**_**3**_^**+**^ and ^**–**^**SNH**_**2**_) pairs. SVD of the Cys S Kβ, Kα, and TFY XAS data matrices all show two major components, which is consistent with there being two unique spectra contributing to the data representing thiol and thiolate, irrespective of amine protonation.

As a result, we can map our fitting analysis from NAC to the Cys data. At pH 6.4 and 13, the fractional concentration of Cys is dominated by **HSNH**_**3**_^**+**^ and ^**–**^**SNH**_**2**_, respectively, but these spectra can still be taken to be generally representative of Cys thiol and thiolate. By fitting the data at intermediate pH points to a linear combination of the pH 6.4 and 13 spectra, we obtain a fractional concentration of thiolate, which here represents the sum concentration of ^**–**^**SNH**_**3**_^**+**^ and ^**–**^**SNH**_**2**_ over the total Cys concentration. We compare these fitted values to the reference curve for total thiolate concentration using the microscopic p*K*_a_’s determined by Benesch and Benesch and find good agreement between the reference curve that accounts for the four microscopic p*K*_a_’s and our fits, particularly for the Kβ and K-edge XAS data, as opposed to the Henderson–Hasselbalch curve for a single p*K*_a_ of 8.33. Similarly, as observed with NAC, the fractional conversions determined from the Kα data track the general trend but exhibit much larger uncertainties.

These results provide a foundation for studying Cys chemistry in proteins using S X-ray spectroscopy and point to two direct applications: (1) to assess Cys thiol p*K*_a_ in protein systems where other techniques are ambiguous and (2) to detect bonding changes at Cys thiolates associated with catalytic activity in cysteine dependent enzymes. In pursuit of the second aim, it is valuable considering to which covalent changes these techniques will be most sensitive. From our results, we find S Kβ XES to be a good diagnostic tool to distinguish thiol and thiolate protonation states in NAC and Cys, comparable to S K-edge XAS. However, these spectroscopies are much less sensitive to distal covalent changes in cysteine, including amine protonation (in Cys) and acetylation (comparing NAC and Cys). Qureshi et al. reported similarly little sensitivity in the S Kβ XES in aliphatic thiols and sulfides outside of ring strain effects in cyclic systems. In comparison, Qureshi et al. found S Kβ XES to be more sensitive to ring substituents in a series of thiophenes, where the S 3p orbitals are involved in a delocalized aromatic system.^34^

As expected, the S Kβ XES is most informative when differentiating a change in S bonding that directly impacts the distribution of S 3p electrons. For Cys, this sensitivity could include S protonation state change, intermediate formation following nucleophilic attack of a Cys thiolate, or potentially changes in the hydrogen bonding network around S in a protein. Cysteine dependent enzymes like isocyanide hydratase (ICH) are good examples where these techniques could elucidate Cys chemistry critical to protein function. ICH catalyzes the hydration of isocyanide substrates to formamides via a thioimidate formed with the active site Cys thiolate, whose reactivity is modulated by a local hydrogen bonding network.^43^ S X-ray spectroscopy could serve as an appropriate probe for studying the chemistry and bonding occurring at the active site Cys as the enzyme proceeds through turnover.

Any such spectral changes may be slight, particularly in time-resolved measurements where only a fraction of the species may be converted at any given time. Even for Cys, where small spectral differences in the lower energy range of the S Kβ XES were predicted by DFT for **HSNH**_**3**_^**+**^ and **HSNH**_**2**_, such differences were not resolved experimentally. High resolution and high signal-to-noise S Kβ XES is thus a priority for studying S chemistry in proteins but presents a current limitation. For our present study, NAC and Cys samples were prepared at 50 mM concentrations, which is well above the solubility limits for many proteins. However, the concentration constraints can be overcome by combining these measurements with serial crystallography experiments on crystalline protein samples and technological developments such as a new multielement von Hamos type tender X-ray spectrometer that is being prepared for use at the Linac Coherent Light Source and Stanford Synchrotron Radiation Lightsource to enable study of lower concentration samples with high signal-to-noise.

In conclusion, we demonstrate the sensitivity of S K line X-ray emission to pH-dependent chemical changes in NAC and Cys. The thiol and thiolate protonation states of NAC and Cys are well distinguished by S Kβ XES which compares favorably with S K-edge XAS. DFT simulation of the VtC transitions provides good agreement with the measured Kβ spectra, allowing us to analyze the valence orbital contributions to these transitions. The distribution of S 3p orbitals between hybrid bonding orbitals and nonbonding orbitals is key for understanding the Kβ differences observed between thiol and thiolate. This also suggests why little difference is observed comparing NAC and Cys data, as covalent changes at the amine do not significantly alter the S 3p electrons. S Kα XES exhibits a small shift (∼0.05 eV) moving from thiol to thiolate that is consistent with increased electron density in the thiolate S 3p orbitals compared to the thiol. Although the data can distinguish this change in the S Kα data, it serves as the least accurate of the three techniques for determining speciation at intermediate pH. Understanding sulfur ionization and bonding is an important challenge common to small molecule and macromolecular samples. We thus show that S K line XES, especially the Kβ line, can be a potent tool to assess physiologically relevant chemical changes at the Cys sulfur. This analysis can be directly applied for assessing Cys S ionization and p*K*_a_ in protein environments, which we will apply to protein samples in future studies. These results also suggest the utility of S Kβ and K-edge XAS in time-resolved studies of cysteine dependent enzymes where active site Cys residues undergo changes in ionization and bonding throughout the catalytic cycle, providing an element specific window into the critical S chemistry in these proteins.

## Supporting Information Available

Materials and experimental methods; description of Cys ionization equilibrium; energy calibration; UV–vis pH titrations; singular value decomposition of data; description and results of fitting procedures for intermediate pH data; computational details and orbital contribution analysis for TDDFT prediction of XANES and VtC prediction of S Kβ XES (PDF)
Transparent Peer Review report available (PDF)
