# Supplementary material for: Determination of Thiol Protonation States by Sulfur X-ray Spectroscopy in Biological Systems
Source: J Phys Chem Lett. 2025 Feb 27;16(9):2401–8. doi: 10.1021/acs.jpclett.4c03247 (PMC11892467; doi:10.1021/acs.jpclett.4c03247)
Supplement: Supplementary file 1 — jz4c03247_si_001.pdf [file jz4c03247_si_001.pdf]

Supporting Information

Determination of Thiol Protonation States by Sulfur X-ray Spectroscopy in Biological Systems

Ryan D. Ribson[1], Alec H. Follmer[2], Jeffrey T. Babicz Jr.[1,3], Victor Sosa Alfaro[1], Ryan G. Hadt[4], Mark S. Hunter[1], Mark A. Wilson[5], Dimosthenis Sokaras[3], Roberto Alonso-Mori*[1]*

[1]Linac Coherent Light Source, SLAC National Accelerator Laboratory, Menlo Park, California 94025, United States;

[2]Department of Chemistry, University of California, Davis, One Shields Avenue, Davis, California 95616, United States;

[3]SSRL, SLAC National Accelerator Laboratory, Menlo Park, California 94025, United States;

[4]Division of Chemistry and Chemical Engineering, Arthur Amos Noyes Laboratory of Chemical Physics, California Institute of Technology, Pasadena, California 91125, United States;

[5]Department of Biochemistry, University of Nebraska, Lincoln, Nebraska 68588, United States;

Corresponding Authors

Roberto Alonso-Mori - Linac Coherent Light Source, SLAC National Accelerator Laboratory, Menlo Park, California 94025, United States; E-mail: robertoa@slac.stanford.edu

Dimosthenis Sokaras - SSRL, SLAC National Accelerator Laboratory, Menlo Park, California 94025, United States; E-mail: dsokaras@slac.stanford.edu

Table of Contents

Experimental Details

Samples………………………………………………… 3

X-ray Spectroscopy………………………………….. 4

BL6-2 Sample Delivery…………………………... 5

Intermediate pH Fitting Procedure…………………... 7

UV-Vis pH Titrations……………………………….... 7

pH Titration Reference Curves………………………. 8

Computational Details

Density Functional Theory…………………………... 12

S Kβ Energy Axis Calibration……………………………….. 13

S Kα Energy Axis Calibration……………………………….. 14

NAC

UV-Vis Titration……………………………………… 15

Kβ XES………………………………………………. 16

Kα XES………………………………………………. 17

Kα RIXS and TFY XAS……………………………… 24

Cys

UV-Vis Titration……………………………………… 30

Kβ XES………………………………………………. 31

Kα XES………………………………………………. 34

Kα RIXS and TFY XAS……………………………... 39

DFT

HSNHAc…………………………………………….. 45

$^{-}$SNHAc…………………………………………… 50

$HSNH_3^+$…………………………………………… 55

$^{-}SNH_2$………………………………………………... 60

$HSNH_2$………………………………………………. 65

$^{-}SNH_3^+$……………………………………………… 70

References…………………………………………………... 76

Experimental Details

Samples

L-cysteine, *N*-acetyl-L-cysteine, glycylglycine, and sodium hydroxide were purchased from Sigma Aldrich and used without further purification. Citric acid monohydrate was purchased from Fisher Scientific and used without further purification.

For the X-ray spectroscopy experiments, sample solutions were prepared immediately before X-ray exposure and data collection. All solutions were prepared in deionized water run through an Elga PURELAB water purification system. Stock solutions of 1 M glycylglycine (132.12 g/mol; 0.05 mol or 6.6 g in 50 mL H_2O), 1 M citric acid (210.14 g/mol; 0.05 mol or 10.5 g in 50 mL H_2O), and 10 N sodium hydroxide (40.0 g/mol; 1 mol or 40.0 g in 100 mL H_2O) were prepared ahead of time. For each sample the goal concentrations were: 50 mM L-cysteine (or *N*-acetyl-L-cysteine), 250 mM glycylglycine, and 250 mM citric acid (yielding 500 mM total buffer concentration).

Each sample was prepared by adding L-cysteine (121.16 g/mol; 2.5 mmol or 302.9 mg) (for *N*-acetyl-L-cysteine: 163.19 g/mol; 2.5 mmol or 408.0 mg) to a 50 mL falcon tube followed by 12.5 mL of 1 M glycylglycine and 12.5 mL of 1 M citric acid monohydrate. 10 N sodium hydroxide was then used to titrate the solution to the desired pH. H_2O was then added to bring the total volume of the solution to 50 mL.

X-ray Spectroscopy

Sulfur XES and XAS spectra were collected at SSRL beamline end-station 6-2a. 6-2a is a wiggler end-station that utilizes a Si(111) double crystal monochromator configured for use with a tender X-ray spectrometer that has been detailed elsewhere.[1] Briefly, this spectrometer is designed around an off-Rowland circle geometry, using a cylindrically bent Si(111) Johansson-type analyzer crystal to collect emitted X-ray emission from the sample position and diffract it onto a CCD area detector, yielding energy-resolved emission spectra after signal processing. By stepping the 6-2 monochromator through an element's absorption edge, full resonant inelastic X-ray scattering (RIXS) planes can be acquired and total fluorescence yield (TFY) or high energy resolution fluorescence detected (HERFD) XAS spectra can be extracted by selective integration over the emission axis. The spectrometer is designed to maintain the sample compartment under He atmosphere, while keeping the spectrometer compartment under vacuum (~1 x 10^{-6} torr). He-filled ionization chambers were located just before and after the sample chamber along the X-ray beam path, providing reference data for incident and transmitted beam intensity, respectively. Non-resonant S Kα and Kβ XES were collected using an incident X-ray energy of 2800 eV. The S K-edge XAS spectra reported herein were derived from S Kα RIXS measurements, scanning the 6-2 monochromator in the energy region from 2463 to 2538 eV with higher point density in the S K-edge XANES region.

The S Kβ XES energy axis was calibrated using elastic scattering measurements off a water liquid jet. The elastic scattering was collected at incident X-ray energies of 2455, 2465, 2475, and 2485 eV and the resulting peaks were fitted to gaussian features, the centers of which were then fit to a first-order polynomial to provide a conversion between pixel and eV. The 6-2 source does not reach low enough energies (cut-off ~2360 eV) in order to calibrate the S Kα energy axis by

this elastic scattering method. In this case, we collected non-resonant S Kα XES on solutions of reference compounds Na_2SO_3 and Na_2SO_4. These reference spectra were fit to the sum of two Lorentzian lineshapes representing the $K\alpha_1$ and $K\alpha_2$ features. The centers of the fitted $K\alpha_1$ peaks were then used in a two point calibration against the reported $K\alpha_1$ positions of Na_2SO_3 and Na_2SO_4 (2308.88 and 2309.12 eV, respectively).[2]

BL6-2 Sample Delivery

The samples were delivered to the X-ray interaction point as a 250 μm liquid jet with sample recirculation. An HPLC pump (Shimadzu LC-40D) was used to transport sample solution from the reservoir to the sample rod within the sample chamber. The sample chamber of the spectrometer is contained in its own He environment. At the end of the sample rod was affixed a custom-machined PEEK compartment surrounding the jet nozzle. The jet nozzle reduces 1/16" tubing through a 250 μm ID fused silica capillary, generating a 250 μm diameter cylindrical Rayleigh jet that is shot vertically down through the interaction point. This PEEK compartment attaches to a ¼" ID catcher tube via a hose barb that can then be fed back into the sample reservoir. The PEEK compartment was fed its own He line, allowing us to use solvent saturated He around the liquid jet to prevent solvent loss without contaminating the outer sheath of He in the larger sample chamber. A slight overpressure of He within the PEEK compartment was also necessary to ensure solvent egress through the compartment outlet and catcher tube back into the sample reservoir.

The sample reservoir is pictured below in Figure S1. We used a cut falcon tube fitted inside a 100 mL GL45 media bottle to reduce needed sample volume. For each sample, roughly 30 mL of solution would be transferred to the falcon tube insert. A four port GL45 cap with threading for IDEX fittings was used for the reservoir and two of these ports were modified by use of a hand

drill to accommodate larger diameter items. One port was used to introduce ⅛” tubing with a ceramic filter head used as the inlet to the HPLC pump. Another port was used to feed the ¼” catcher tubing from the sample chamber into the reservoir, allowing sample to be recollected and recirculated during the experiment. Another port was used as a He vent to prevent overpressurization of the system. The last port was then used to insert a pH probe to monitor the pH in the sample reservoir over the course of the experiment. This allowed us to keep each sample largely under He atmosphere during the ~6 hours of data collection.

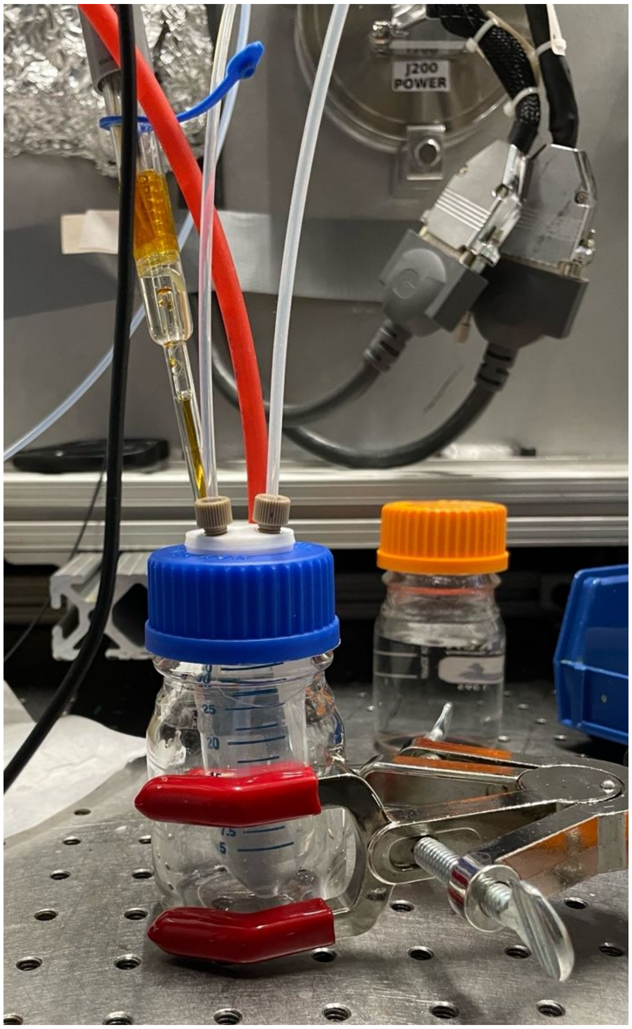

Figure S1. Picture of the sample reservoir used during the X-ray spectroscopic experiments at BL6-2.

We note that it was important to remove any brass fittings/connectors from the experimental set up as the cysteine thiolate (high pH) was capable of stripping Cu from the brass, leading to a yellow coloration of the solution. The use of plastic fittings prevented any such contamination (the interior of the HPLC pump used is steel).

Intermediate pH Fitting Procedure

For both NAC and Cys, the spectra acquired at low pH (~6) and high pH (~13) were taken as representative basis spectra for thiol and thiolate, respectively, and used to fit the intermediate pH data as the following linear combination:

$$\boldsymbol{A} = (1 - \beta) \cdot \boldsymbol{\epsilon_{SH}} + \beta \cdot \boldsymbol{\epsilon_{S^-}} \tag{1}$$

Here, $\boldsymbol{A}$ is the vector representation of a given spectrum to be fit, $\boldsymbol{\epsilon_{SH}}$ is the thiol basis spectrum, $\boldsymbol{\epsilon_{S^-}}$ is the thiolate basis spectrum, and the fitted parameter β represents the fraction of molecules converted to thiolate.

UV-vis pH Titrations

For the UV-vis titration curves, separate aqueous buffered solutions of 10 mM Gly-Gly and 10 mM citric acid were pH'd with 10 N sodium hydroxide solution to the desired pH value (6.2, 7.4, 8.5, 9, 9.5, 9.9, 10.5, 11.6, and 13). 5 mL of the buffer solution was then taken and 25 µL of 20 mM NAC (or Cys) stock solution was added to produce a final NAC (or Cys) concentration of 100 µM. UV-vis spectra were taken in a 10 mm pathlength quartz cuvette on a Thermo Scientific NanoDrop One^{C} spectrophotometer. Each pH point was blanked with its

corresponding buffer. The absorbance at 232 nm was used to determine the fraction of thiolate for NAC. For Cys, as previously reported, the absorption maximum of this band shifts from 232 nm below pH 10 to 236 nm above pH 10. As such, the absorbance at 232 nm is taken for pH values below 10 and the absorbance at 236 nm is taken for pH values above 10. To calculate the fractional conversion (f), the minimum (a_{min}) and maximum (a_{max}) absorbance values at these wavelengths are taken as the floor and ceiling values respectively and the absorbance taken at each pH point (a_i) is then used to calculate f accordingly:

$$f = \frac{a_i - a_{min}}{a_{max} - a_{min}} \tag{2}$$

pH Titration Reference Curves

The thiol/thiolate equilibrium of NAC follows Henderson-Hasselbalch behavior with a reported pK_a of 9.5. The Henderson-Hasselbalch equation as presented below is used for the NAC reference curves of fraction of thiolate vs pH in the main text:

$$\frac{[RS^-]}{[RSH] + [RS^-]} = \frac{10^{pH-pK_a}}{1 + 10^{pH-pK_a}} \tag{3}$$

The thiol/thiolate equilibrium in Cys does not follow the Henderson-Hasselbalch equation as the amine group undergoes protonation/deprotonation in a similar pH range as the thiol. Instead, the four protonation states $\mathbf{HSNH_3^+}$, $\mathbf{HSNH_2}$, $\mathbf{^-SNH_3^+}$, and $\mathbf{^-SNH_2}$ must be considered as in Figure

S2.

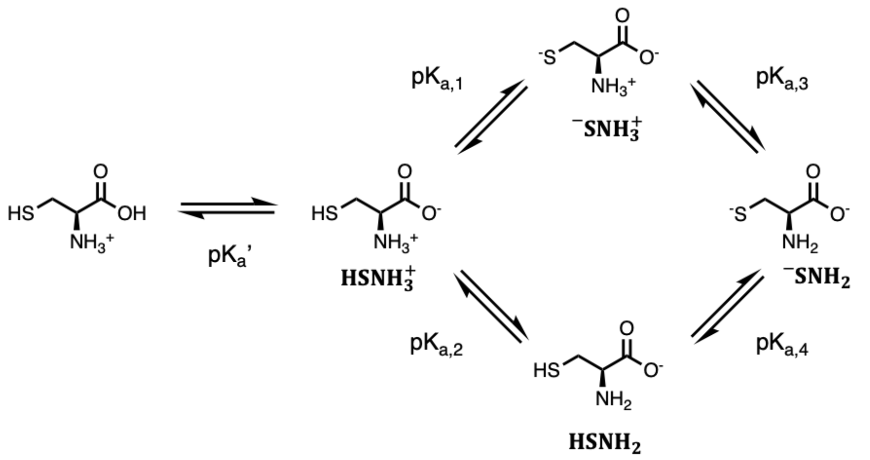

$$K_{a,1} = \frac{[H^+][{}^-SNH_3^+]}{[HSNH_3^+]}$$

$$K_{a,2} = \frac{[H^+][HSNH_2]}{[HSNH_3^+]}$$

$$K_{a,3} = \frac{[H^+][{}^-SNH_2]}{[{}^-SNH_3^+]}$$

$$K_{a,4} = \frac{[H^+][{}^-SNH_2]}{[HSNH_2]}$$

Figure S2. Cys protonation equilibria and relevant equilibrium constants.

The carboxylic acid deprotonation exhibits a pK_a (pK_a') of 1.71 and will be fully deprotonated in the pH 6 - 13 region examined in this study. The reported microscopic pK_a's for the remaining equilibria are $pK_{a,1} = 8.53$, $pK_{a,2} = 8.86$, $pK_{a,3} = 10.03$, and $pK_{a,4} = 10.36$.[3,4] As outlined by Benesch and Benesch, the total thiolate concentration [RS^-] = [$^-$**SNH_3^+**] + [$^-$**SNH_2**] as a fraction of the total sample concentration can be calculated as follows.

To put this equation in terms of pH and pK_a values, we can use the equilibrium constants $K_{a,1}$, $K_{a,2}$, $K_{a,3}$, and $K_{a,4}$ and put everything in terms of a common concentration [$HSNH_2$].

$$\frac{[RS^-]}{[Cys]_{total}} = \frac{[{}^-SNH_3^+] + [{}^-SNH_2]}{[HSNH_3^+] + [{}^-SNH_3^+] + [HSNH_2] + [{}^-SNH_2]} \quad (4)$$

$$\frac{K_{a,1}}{K_{a,2}} = \frac{[H^+][{}^-SNH_3^+]}{[HSNH_3^+]} \cdot \frac{[HSNH_3^+]}{[H^+][HSNH_2]} = \frac{[{}^-SNH_3^+]}{[HSNH_2]} \quad (5)$$

$$[HSNH_3^+] = \frac{[H^+]}{K_{a,2}} \cdot [HSNH_2] \quad (6)$$

$$\left[{}^{-}SNH_3^+\right] = \frac{K_{a,1}}{K_{a,2}} \cdot [HSNH_2] \tag{7}$$

$$[{}^{-}SNH_2] = \frac{K_{a,4}}{[H^+]} \cdot [HSNH_2] \tag{8}$$

Plugging these terms into our equation for total thiolate concentration allows us to cancel the $[HSNH_2]$ terms.

$$\frac{[RS^-]}{[Cys]_{total}} = \frac{\frac{K_{a,1}}{K_{a,2}} \cdot [HSNH_2] + \frac{K_{a,4}}{[H^+]} \cdot [HSNH_2]}{\frac{[H^+]}{K_{a,2}} \cdot [HSNH_2] + \frac{K_{a,1}}{K_{a,2}} \cdot [HSNH_2] + [HSNH_2] + \frac{K_{a,4}}{[H^+]} \cdot [HSNH_2]} \tag{9}$$

The equilibrium constants and $[H^+]$ can then be converted into pK_a and pH terms.

$$\frac{[RS^-]}{[Cys]_{total}} = \frac{\frac{K_{a,1}}{K_{a,2}} + \frac{K_{a,4}}{[H^+]}}{\frac{[H^+]}{K_{a,2}} + \frac{K_{a,1}}{K_{a,2}} + 1 + \frac{K_{a,4}}{[H^+]}} \tag{10}$$

$$\frac{[RS^-]}{[Cys]_{total}} = \frac{\frac{10^{-pK_{a,1}}}{10^{-pK_{a,2}}} + \frac{10^{-pK_{a,4}}}{10^{-pH}}}{\frac{10^{-pH}}{10^{-pK_{a,2}}} + \frac{10^{-pK_{a,1}}}{10^{-pK_{a,2}}} + 1 + \frac{10^{-pK_{a,4}}}{10^{-pH}}} \tag{11}$$

This yields the final equation used for the reference curve in the main text.

$$\frac{[RS^-]}{[Cys]_{total}} = \frac{10^{pK_{a,2}-pK_{a,1}} + 10^{pH-pK_{a,4}}}{10^{pK_{a,2}-pH} + 10^{pK_{a,2}-pK_{a,1}} + 1 + 10^{pH-pK_{a,4}}} \tag{12}$$

The concentrations of each individual protonation state can be calculated as thus:

$$\frac{[HSNH_3^+]}{[Cys]_{total}} = \frac{10^{pK_{a,2}-pH}}{10^{pK_{a,2}-pH} + 10^{pK_{a,2}-pK_{a,1}} + 1 + 10^{pH-pK_{a,4}}} \tag{13}$$

$$\frac{[^{-}SNH_3^+]}{[Cys]_{total}} = \frac{10^{pK_{a,2}-pK_{a,1}}}{10^{pK_{a,2}-pH} + 10^{pK_{a,2}-pK_{a,1}} + 1 + 10^{pH-pK_{a,4}}} \tag{14}$$

$$\frac{[HSNH_2]}{[Cys]_{total}} = \frac{1}{10^{pK_{a,2}-pH} + 10^{pK_{a,2}-pK_{a,1}} + 1 + 10^{pH-pK_{a,4}}} \tag{15}$$

$$\frac{[^{-}SNH_2]}{[Cys]_{total}} = \frac{10^{pH-pK_{a,4}}}{10^{pK_{a,2}-pH} + 10^{pK_{a,2}-pK_{a,1}} + 1 + 10^{pH-pK_{a,4}}} \tag{16}$$

Computational Details

Density Functional Theory

Density functional theory (DFT) calculations were performed using the ORCA quantum chemistry package (version 4.2.1).[5] DFT geometry optimizations were first carried out and optimized structures were confirmed via vibrational frequency calculations yielding no imaginary frequency modes. The valence-to-core (VTC) XES spectra were simulated from the ground state optimized DFT results. The XAS near-edge region was simulated using time-dependent DFT (TD-DFT) from the geometry-optimized DFT results. All calculations employed the B3LYP functional, the Douglas-Kroll-Hess (DKH2) scalar relativistic Hamiltonian, and the available Ahlrichs def2-TZVPP basis set recontracted for use with the DKH Hamiltonian (DKH-def2-TZVPP).[6] The conductor-like polarizable continuum model (CPCM) was implemented, selecting water as the solvent model (dielectric constant 80.4). The "orca_mapspc" function was used to generate stick spectra of the simulated XES/XAS. For comparison with the experimental spectra, line broadening was applied to the theoretical stick spectra using a Voigtian lineshape and the theoretical spectra were shifted to align in energy with the experimental results. In general, we found that different shift values were required for the different species bearing distinct formal charges. We note the applied shift value for each case where appropriate.

S Kβ Energy Axis Calibration

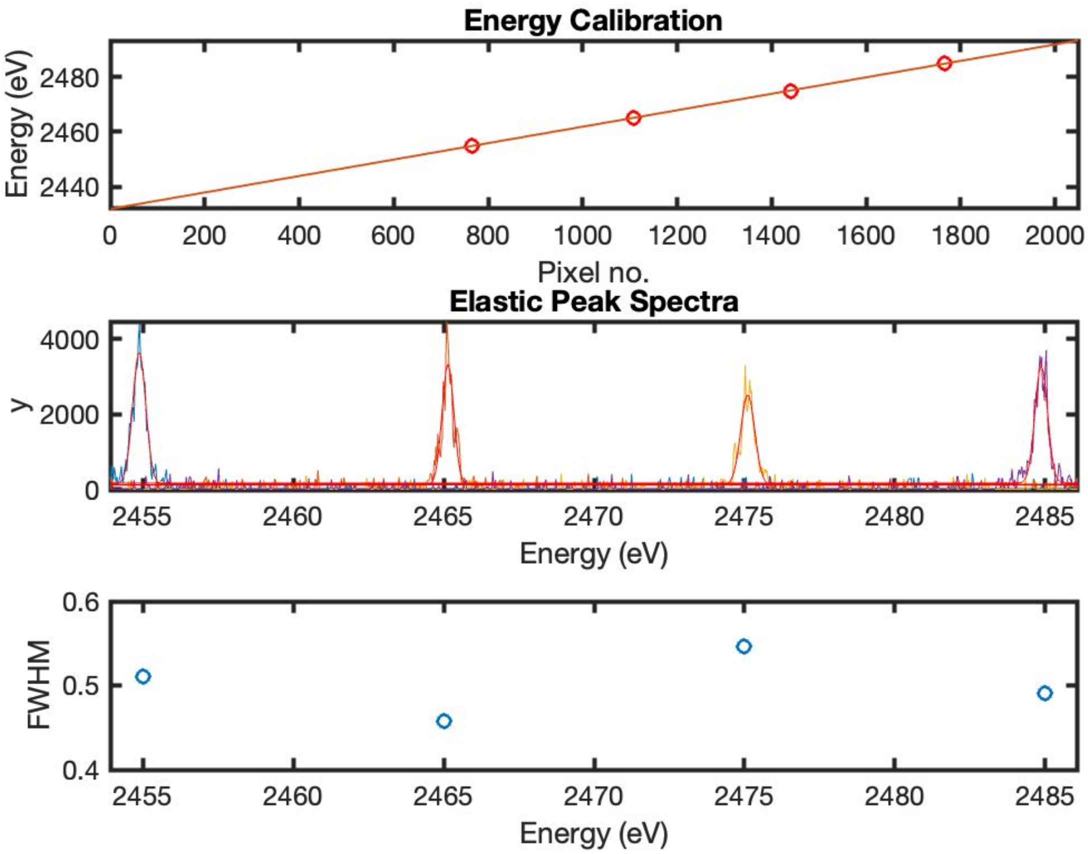

Figure S3. Elastic scattering measurements taken at four incident energies (2455, 2465, 2475, and 2485 eV) for calibration of the energy dispersive axis of the detector in the S Kβ spectrometer geometry. The elastic scattering peaks are fitted to a gaussian function whose centroid pixel position is used for the linear calibration curve. The mean FWHM of the fitted gaussians is 0.5 eV.

S Kα Energy Axis Calibration

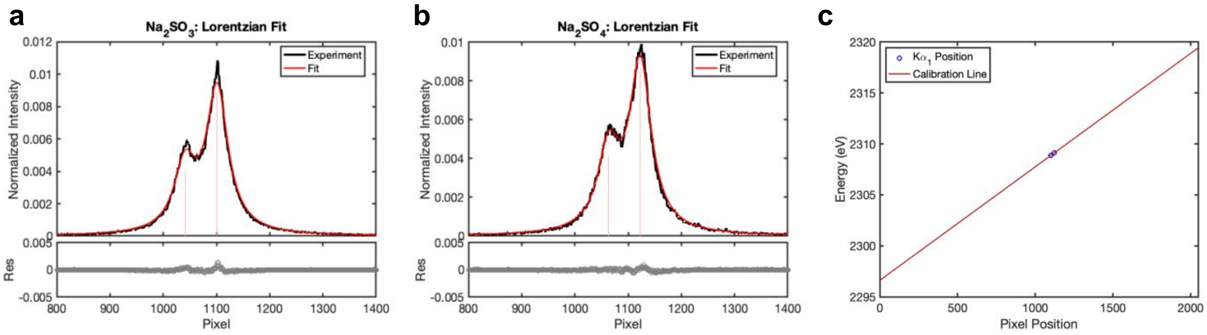

Figure S4. The S Ka energy is too low for the BL6-2 monochromator, so we cannot use elastic scattering to calibrate the Ka energy axis. We collected Ka XES on (a) Na_2SO_3 and (b) Na_2SO_4. The spectra were fit to a model of two Lorentzian functions with fixed linewidths of 0.522 eV (the 1s core hole lifetime of S).[7] (c) The fitted centers of the Ka_1 (given in the table in pixel) were used to calibrate the energy axis against the reported values (Na_2SO_3 Ka_1: 2308.88 eV, Na_2SO_4 Ka_2: 2309.12).[2]

Table S1. Fitted pixel positions of the $K\alpha_1$ and $K\alpha_2$ lines of Na_2SO_3 and Na_2SO_4 used in the S Kα energy axis calibration.

	Na_2SO_3	Na_2SO_4
$K\alpha_1$ (pixel position)	1101	1123
$K\alpha_2$ (pixel position)	1041	1064

NAC

UV-Vis Titration

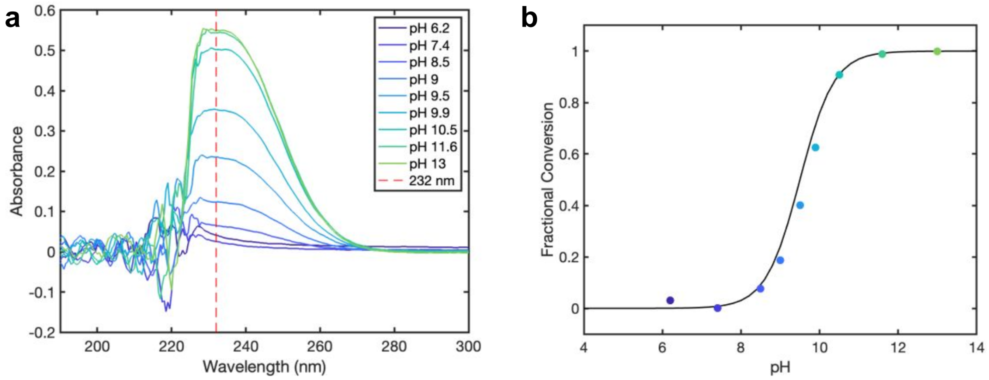

Figure S5. (a) UV-vis absorption spectra of 100 µM NAC in 10 mM Gly-Gly, 10 mM citric acid buffer at varying pH values between 6 and 13. The ma ximum absorbance of the thiolate absorption peak is taken at 232 nm as highlighted by the dashed red line. (b) The calculated fractional conversion to thiolate at each pH point overlaid against the Henderson-Hasselbalch curve for pK_a = 9.5.

NAC Kβ

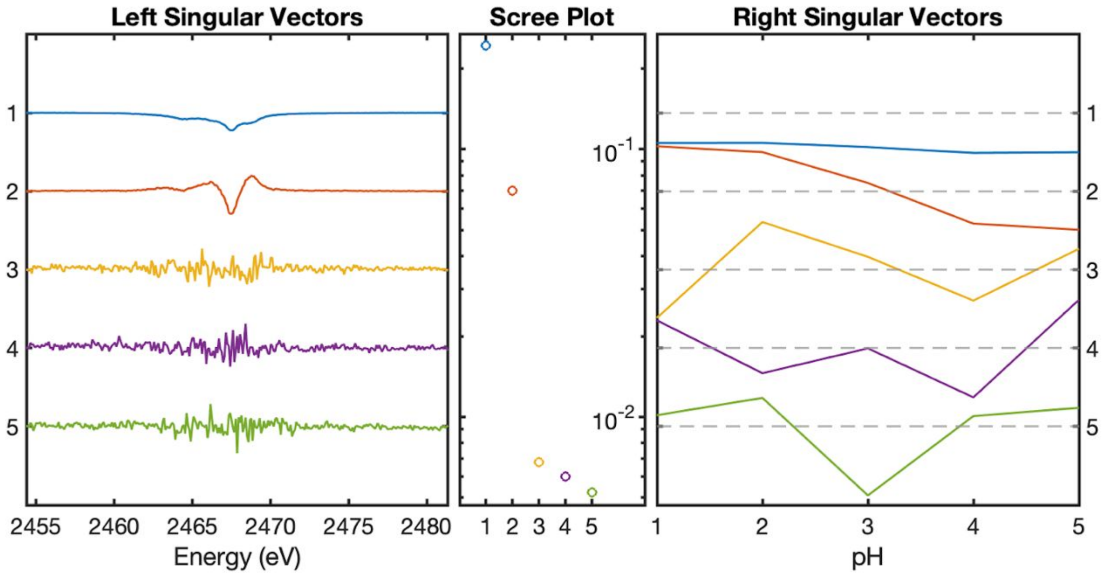

Figure S6. Singular value decomposition performed on the Cys S Kβ XES spectra as a function of pH. Two of the left singular vectors (function of photon energy) have significant structure and correspondingly significant singular values as represented in the scree plot. This is consistent with the thiol and thiolate spectra contributing to the data across the pH values studied.

NAC Kβ - fits of intermediate pH

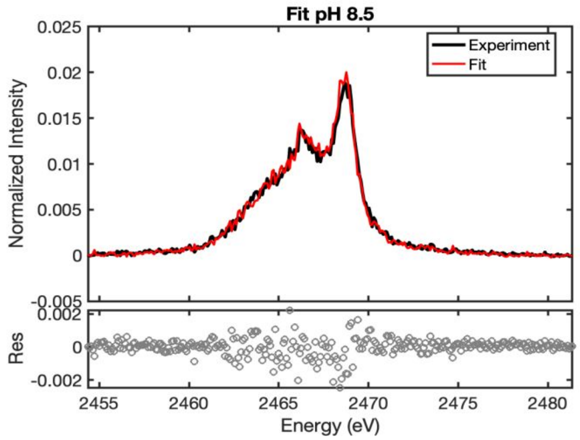

Figure S7. Fit of NAC S Kβ XES spectrum at pH 8.5 overlaid on experimental data with residuals plotted.

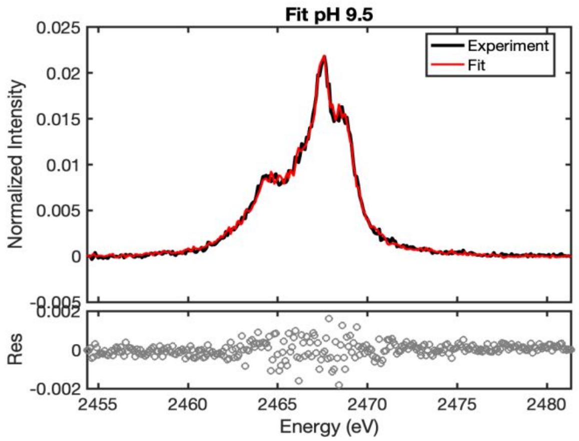

Figure S8. Fit of NAC S Kβ XES spectrum at pH 9.5 overlaid on experimental data with residuals plotted.

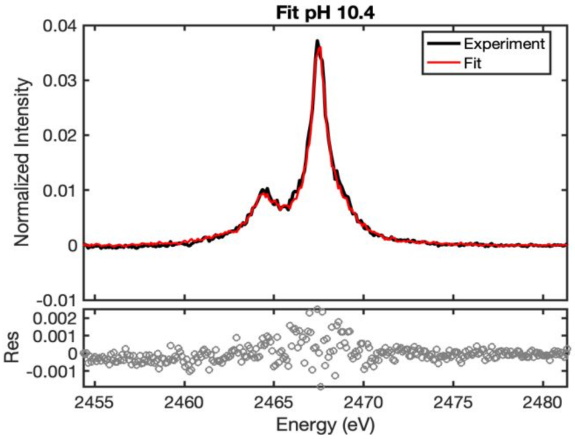

Figure S9. Fit of NAC S Kβ XES spectrum at pH 10.4 overlaid on experimental data with residuals plotted.

NAC Kα Lorentzian Fitting

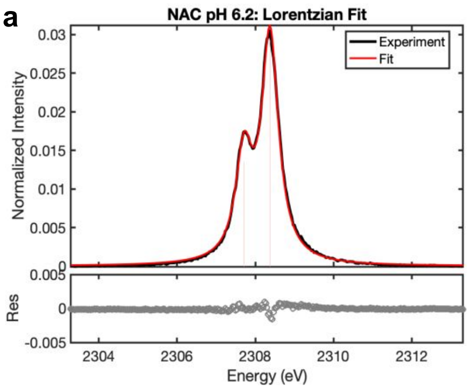

Figure S10. The NAC S Kα XES spectra at **(a)** pH 6.2 (thiol) and **(b)** pH 13 (thiolate) were fit to a sum of two Lorentzian curves with fixed linewidths set to the S 1s core hole lifetime broadening (1.26 fs, 0.52 eV) to extract center positions of the $K\alpha_1$ and $K\alpha_2$ features.

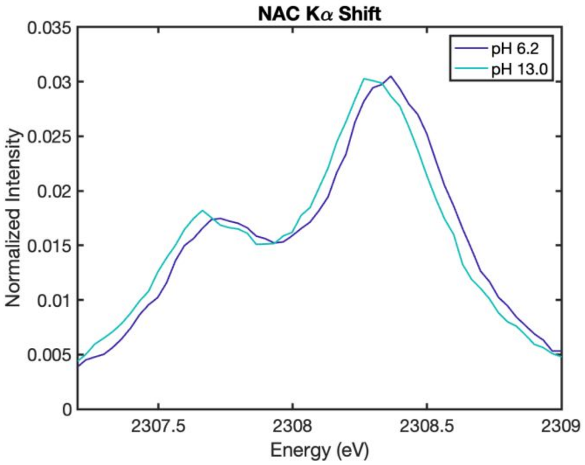

Figure S11. An overlay of the NAC Kα XES spectra at pH 6.2 and 13 to better visualize the shift in Kα energy.

Table S2. Fitted energy positions of the NAC Kα_1 and Kα_2 lines (errors represent 95% confidence intervals from the fit) and tabulated absolute energy shift (Δ) from pH 6.4 (higher energy) to pH 13 (lower energy).

pH	Kα_1 (eV)	Kα_2 (eV)
6.2	2308.374 ± 0.002	2307.710 ± 0.004
13.0	2308.326 ± 0.002	2307.661 ± 0.004
Δ	0.048 ± 0.004	0.049 ± 0.008

NAC Kα - SVD

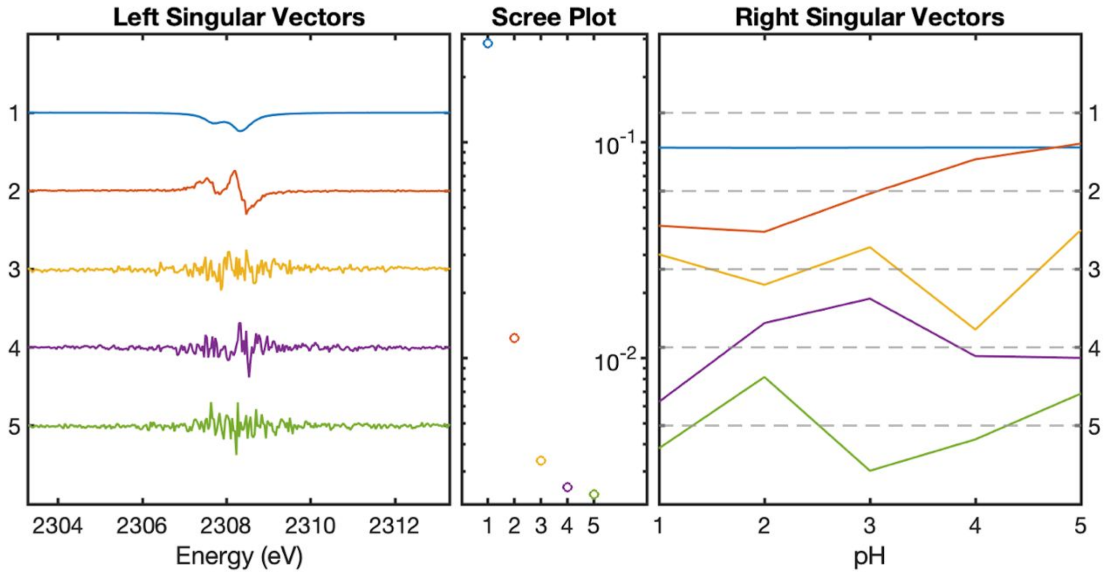

Figure S12. Singular value decomposition performed on the NAC S Kα XES spectra as a function of pH. Two of the left singular vectors (function of photon energy) have significant structure and correspondingly significant singular values as represented in the scree plot. This is consistent with the thiol and thiolate spectra contributing to the data across the pH values studied.

NAC Kα - fits of intermediate pH

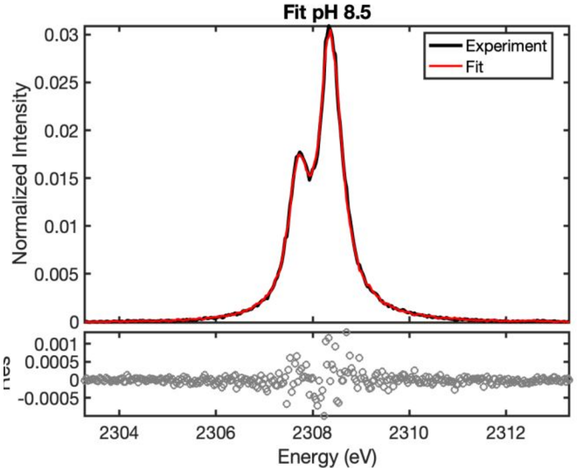

Figure S13. Fit of NAC S Kα XES spectrum at pH 8.5 overlaid on experimental data with residuals plotted.

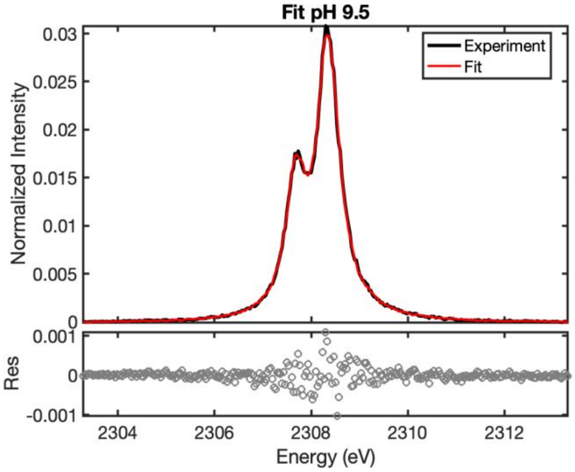

Figure S14. Fit of NAC S Kα XES spectrum at pH 9.5 overlaid on experimental data with residuals plotted.

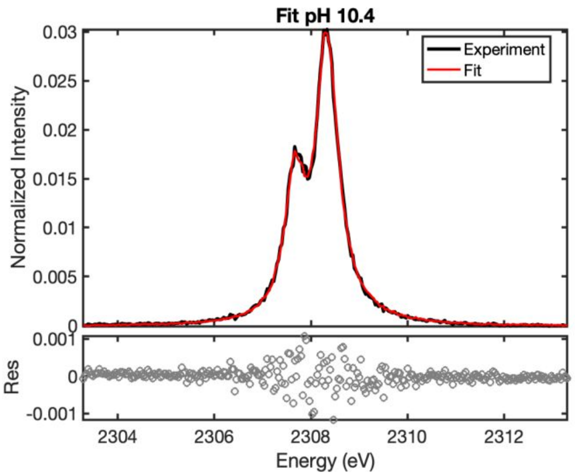

Figure S15. Fit of NAC S Kα XES spectrum at pH 10.4 overlaid on experimental data with residuals plotted.

NAC Kα RIXS/XAS

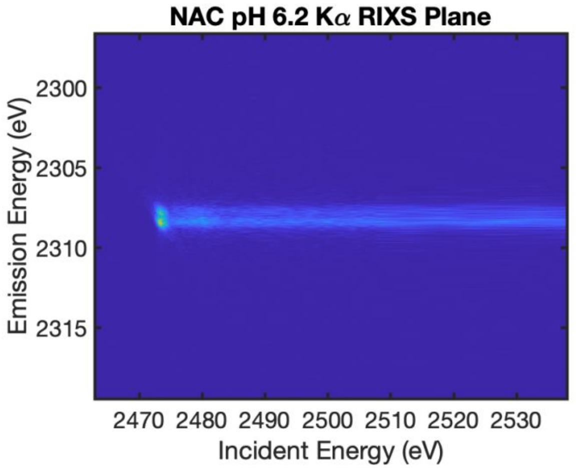

Figure S16. NAC Kα RIXS plane at pH 6.2 as a function of incident X-ray energy and emission energy.

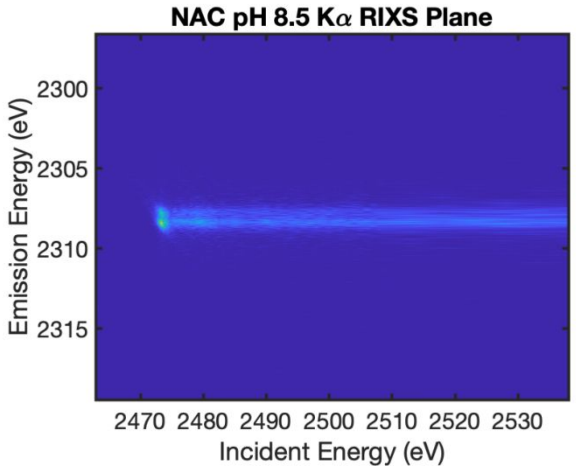

Figure S17. NAC Kα RIXS plane at pH 8.5 as a function of incident X-ray energy and emission energy.

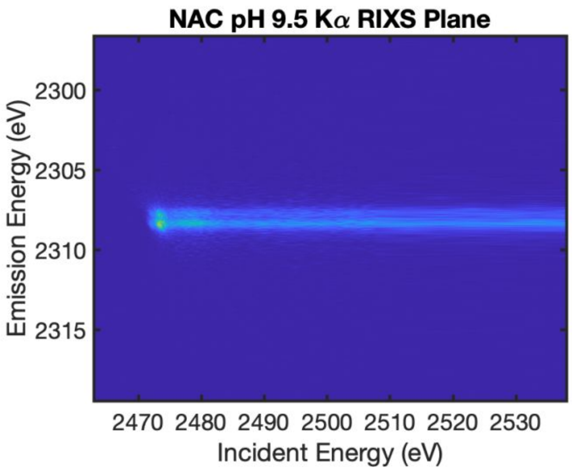

Figure S18. NAC Kα RIXS plane at pH 9.5 as a function of incident X-ray energy and emission energy.

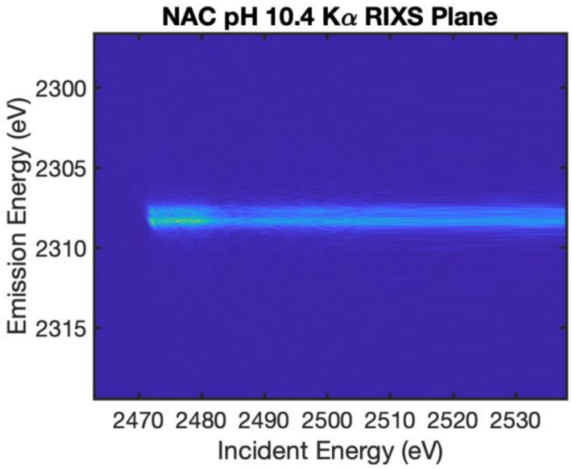

Figure S19. NAC Kα RIXS plane at pH 10.4 as a function of incident X-ray energy and emission energy.

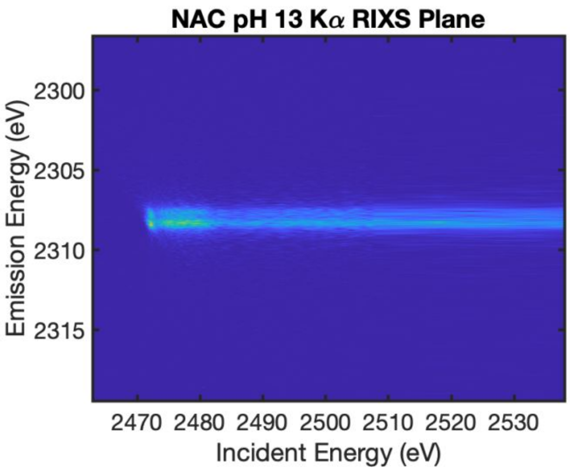

Figure S20. NAC Kα RIXS plane at pH 13 as a function of incident X-ray energy and emission energy.

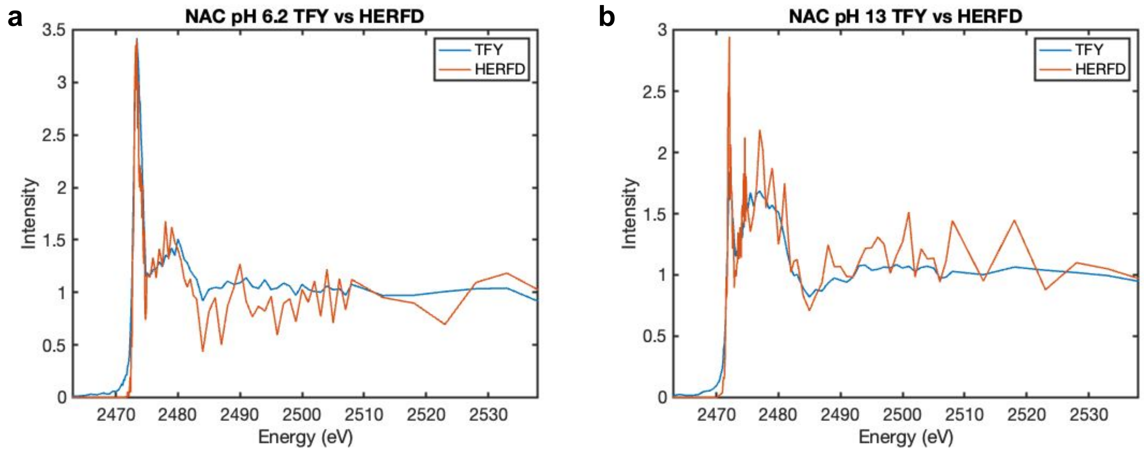

Figure S21. NAC TFY XAS vs HERFD (integrated over $K\alpha_1$ maximum) XAS calculated from the Kα RIXS planes for **(a)** pH 6.2 and **(b)** pH 13.

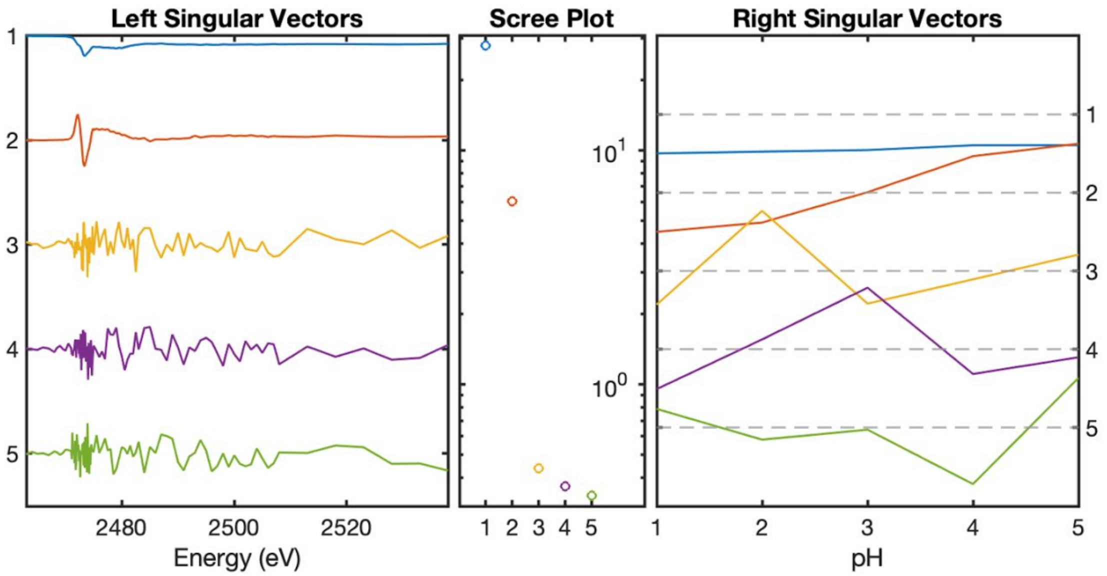

Figure S22. Singular value decomposition performed on the NAC S K-edge TFY XAS spectra as a function of pH. Two of the left singular vectors (function of photon energy) have significant structure and correspondingly significant singular values as represented in the scree plot.

NAC TFY XAS - fits of intermediate pH

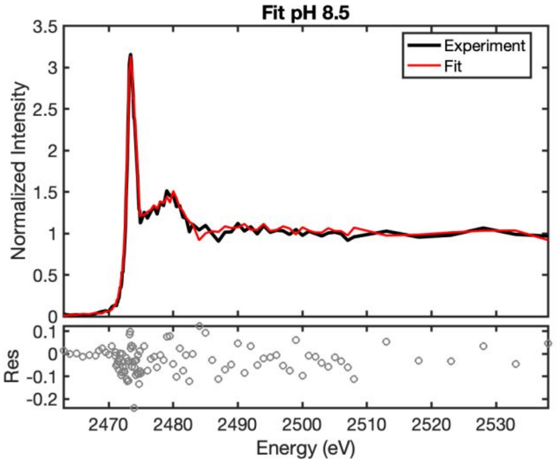

Figure S23. Fit of NAC S K-edge TFY XAS spectrum at pH 8.5 overlaid on experimental data with residuals plotted.

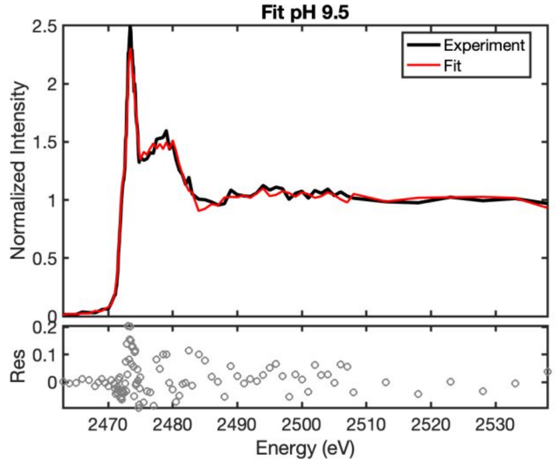

Figure S24. Fit of NAC S K-edge TFY XAS spectrum at pH 9.5 overlaid on experimental data with residuals plotted.

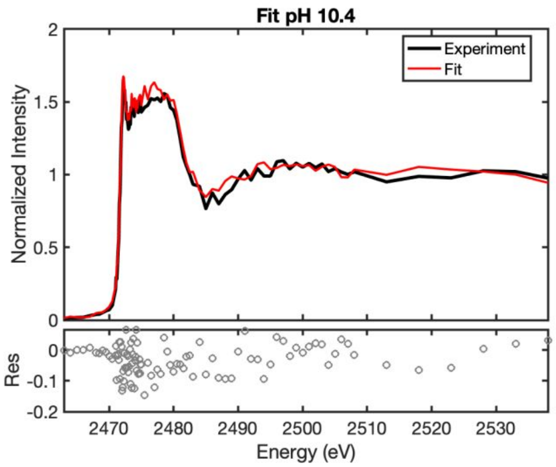

Figure S25. Fit of NAC S K-edge TFY XAS spectrum at pH 10.4 overlaid on experimental data with residuals plotted.

Cys

UV-vis Titration

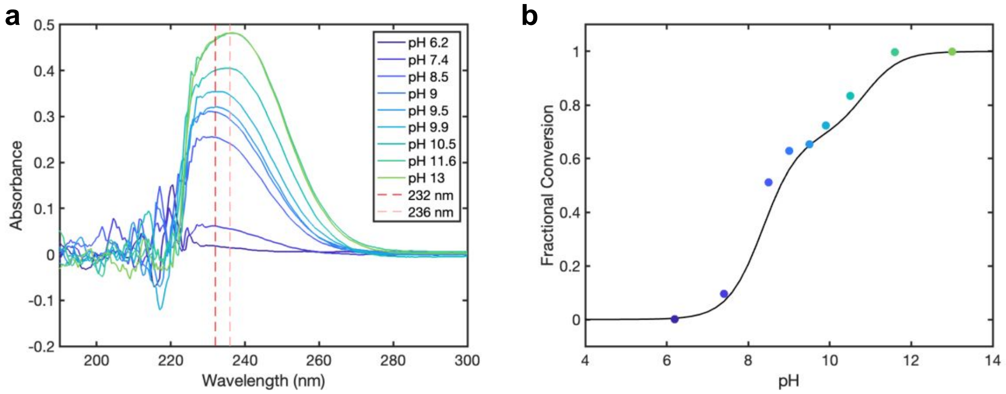

Figure S26 (a) UV-vis absorption spectra of 100 μM Cys in 10 mM Gly-Gly, 10 mM citric acid buffer at varying pH values between 6 and 13. The maximum absorbance of the thiolate absorption peak is taken at 232 nm (pH < 10) and 236 nm (pH > 10) as highlighted by the dashed red and pink lines **(b)** The calculated fractional conversion to thiolate at each pH point overlaid against the theoretical curve calculated from the four microscopic pK_a's reported for Cys.

Cys Kb

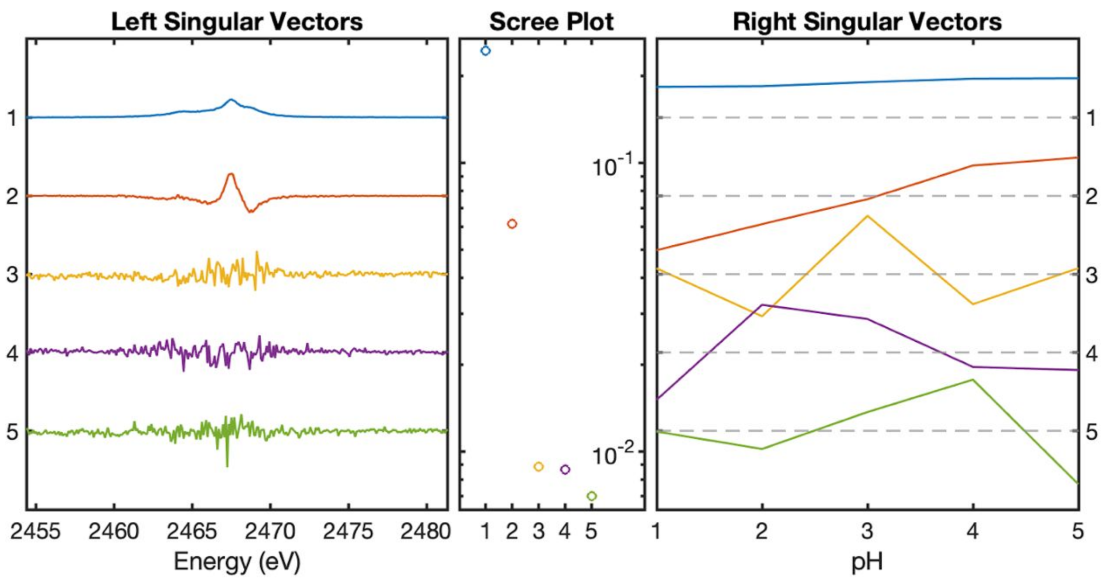

Figure S27. Singular value decomposition performed on the Cys S Kβ XES spectra as a function of pH. Two of the left singular vectors (function of photon energy) have significant structure and correspondingly significant singular values as represented in the scree plot.

Cys Kb - fits of intermediate pH

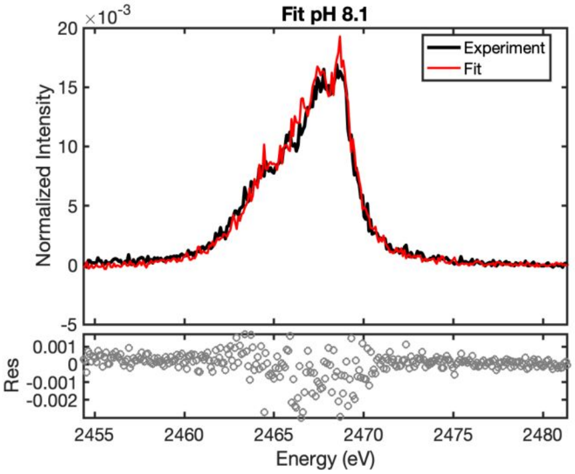

Figure S28. Fit of Cys S Kβ XES spectrum at pH 8.1 overlaid on experimental data with residuals plotted.

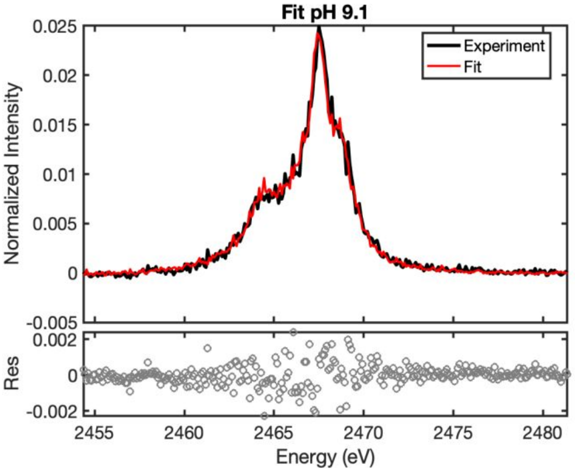

Figure S29. Fit of Cys S Kβ XES spectrum at pH 9.1 overlaid on experimental data with residuals plotted.

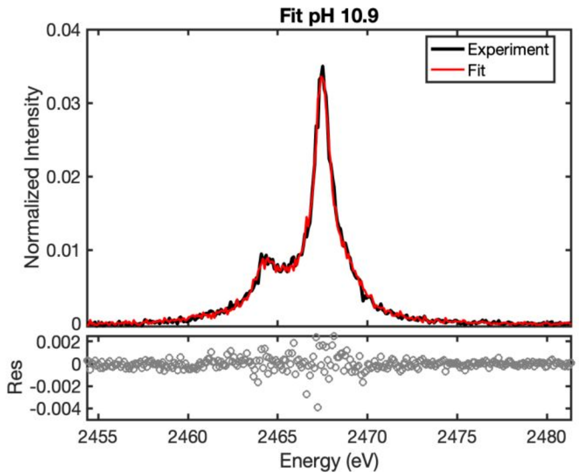

Figure S30. Fit of Cys S Kβ XES spectrum at pH 10.9 overlaid on experimental data with residuals plotted.

Cys Kα Lorentzian Fitting

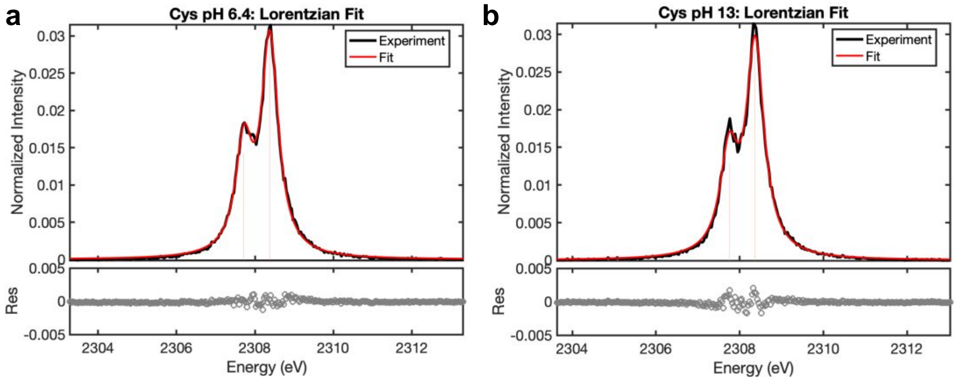

Figure S31. The Cys S Kα XES spectra at **(a)** pH 6.4 (thiol) and **(b)** pH 13 (thiolate) were fit to a sum of two Lorentzian curves with fixed linewidths set to the S 1s core hole lifetime broadening (1.26 fs, 0.52 eV) to extract center positions of the Kα_1 and Kα_2 features.

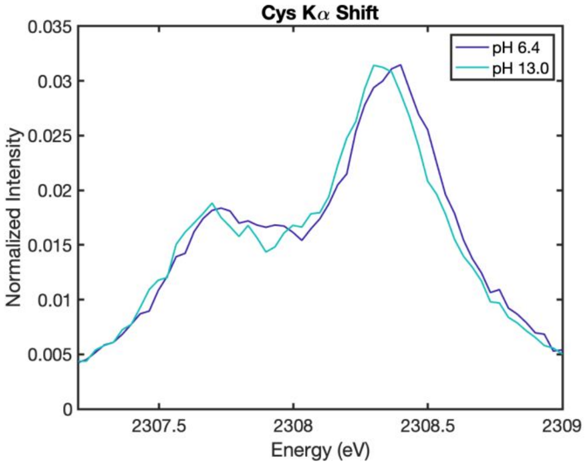

Figure S32. An overlay of the Cys Kα XES spectra at pH 6.4 and 13 to better visualize the shift in Kα energy.

Table S3. Fitted energy positions of the Cys Kα_1 and Kα_2 lines (errors represent 95% confidence intervals from the fit) and tabulated absolute energy shift (Δ) from pH 6.4 (higher energy) to pH 13 (lower energy).

pH	Kα_1 (eV)	Kα_2 (eV)
6.4	2308.379 ± 0.002	2307.716 ± 0.004
13.0	2308.344 ± 0.003	2307.677 ± 0.005
Δ	0.035 ± 0.005	0.039 ± 0.009

Cys Kα - SVD

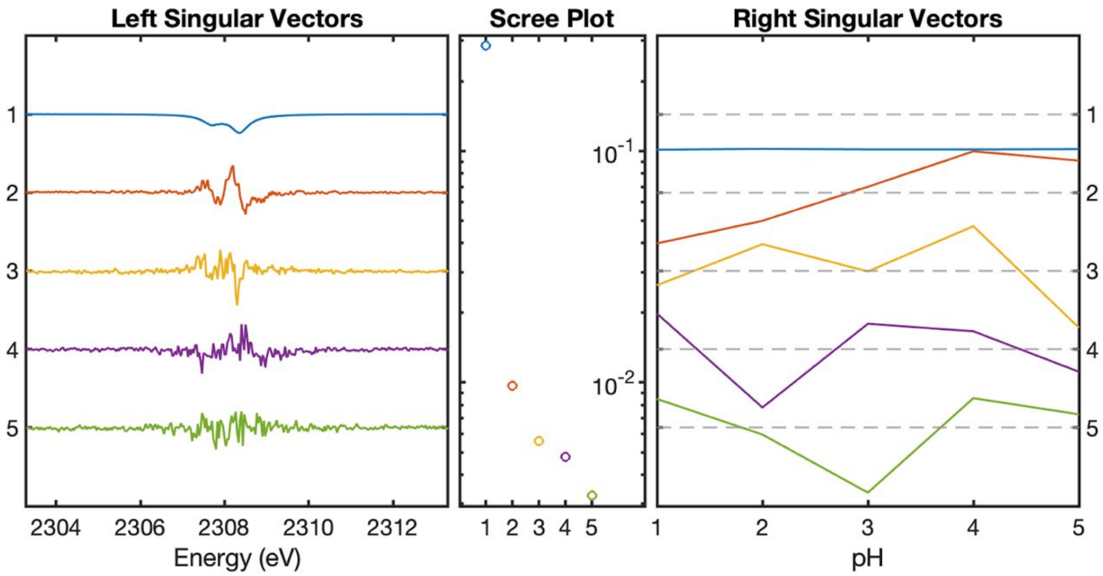

Figure S33. Singular value decomposition performed on the Cys S Kα spectra as a function of pH. Two of the left singular vectors (function of photon energy) have significant structure and correspondingly significant singular values as represented in the scree plot.

Cys Kα - fits of intermediate pH

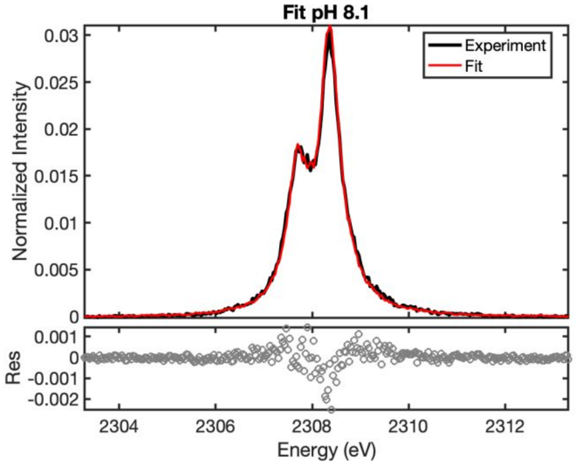

Figure S34. Fit of Cys S Kα XES spectrum at pH 8.1 overlaid on experimental data with residuals plotted.

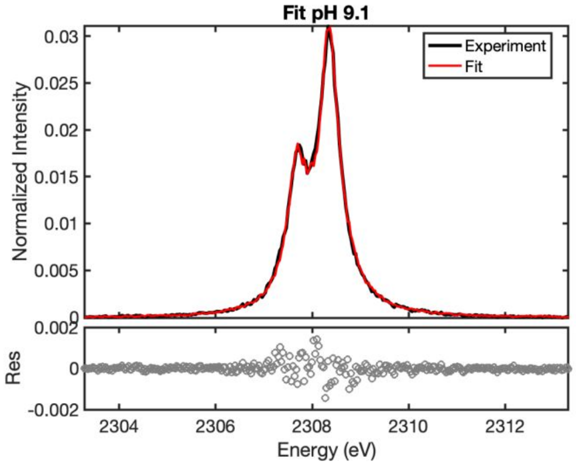

Figure S35. Fit of Cys S Kα XES spectrum at pH 9.1 overlaid on experimental data with residuals plotted.

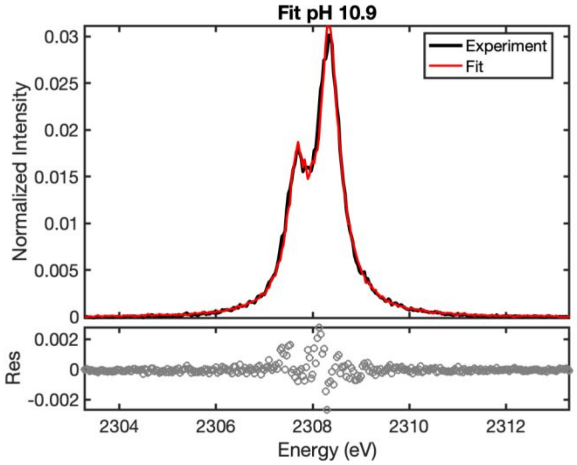

Figure S36. Fit of Cys S Kα XES spectrum at pH 9.1 overlaid on experimental data with residuals plotted.

Cys Kα RIXS/XAS

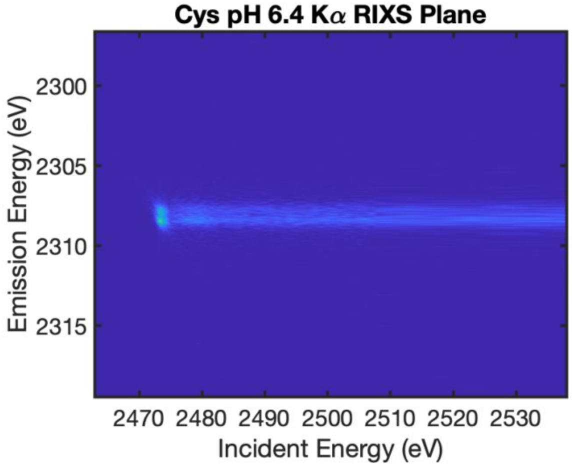

Figure S37. Cys Kα RIXS plane at pH 6.4 as a function of incident X-ray energy and emission energy.

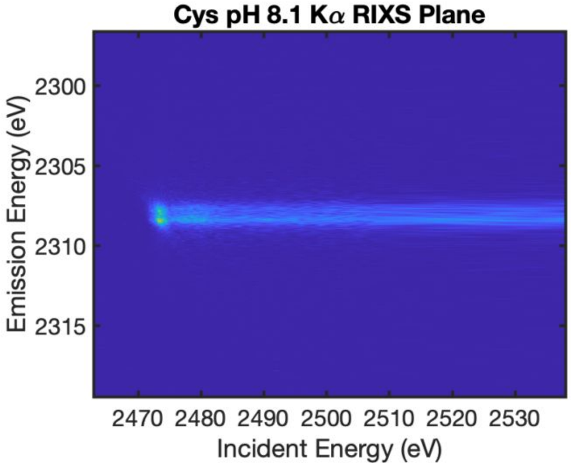

Figure S38. Cys Kα RIXS plane at pH 8.1 as a function of incident X-ray energy and emission energy.

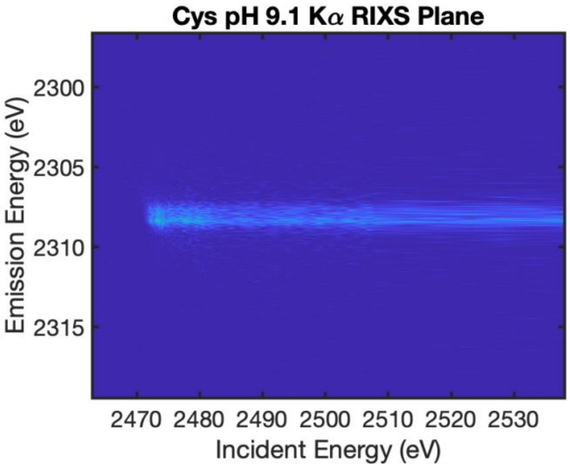

Figure S39. Cys Kα RIXS plane at pH 9.1 as a function of incident X-ray energy and emission energy.

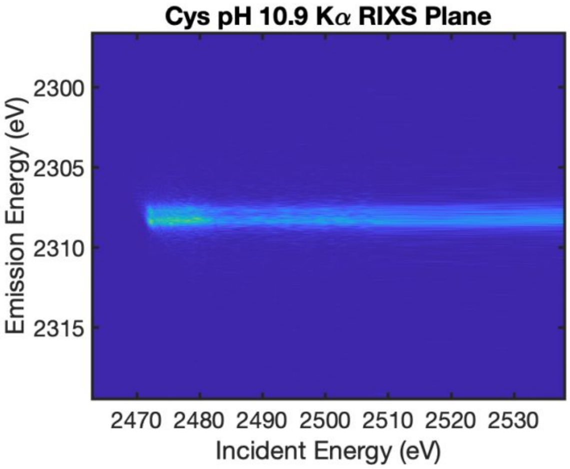

Figure S40. Cys Kα RIXS plane at pH 10.9 as a function of incident X-ray energy and emission energy.

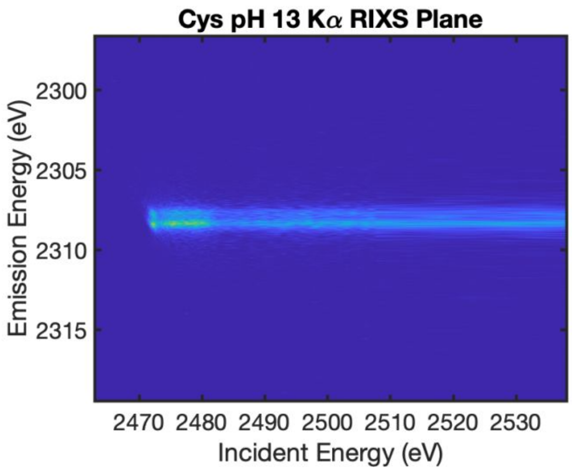

Figure S41. Cys Kα RIXS plane at pH 13 as a function of incident X-ray energy and emission energy.

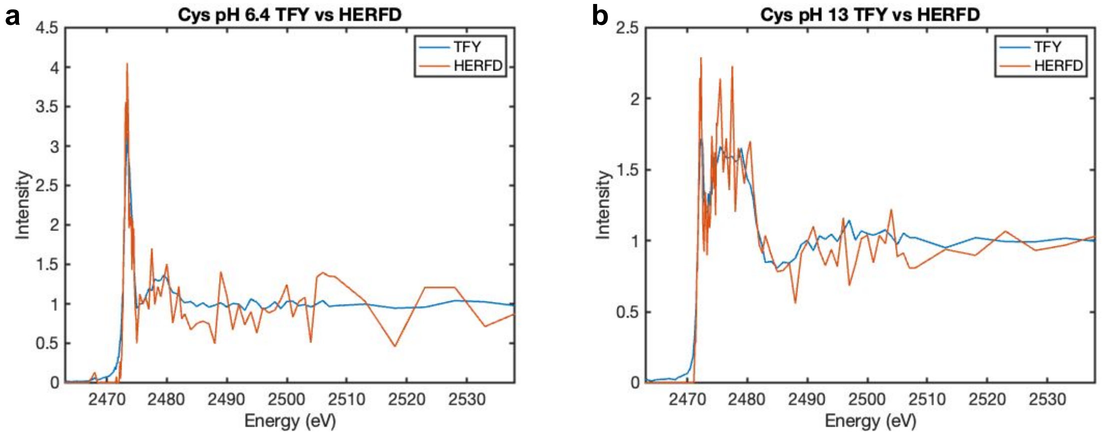

Figure S42. Cys TFY XAS vs HERFD XAS calculated from the Kα RIXS planes for pH 6.4 and pH 13.

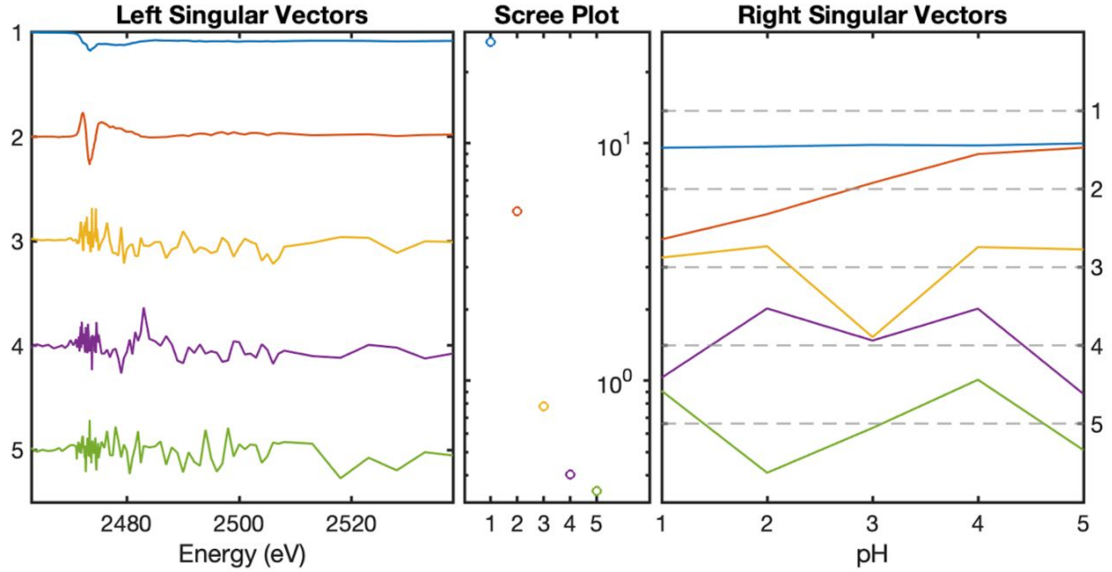

Figure S43. Singular value decomposition performed on the Cys S K-edge TFY XAS spectra as a function of pH. Two of the left singular vectors (function of photon energy) have significant structure and correspondingly significant singular values as represented in the scree plot.

Cys TFY XAS - fits of intermediate pH

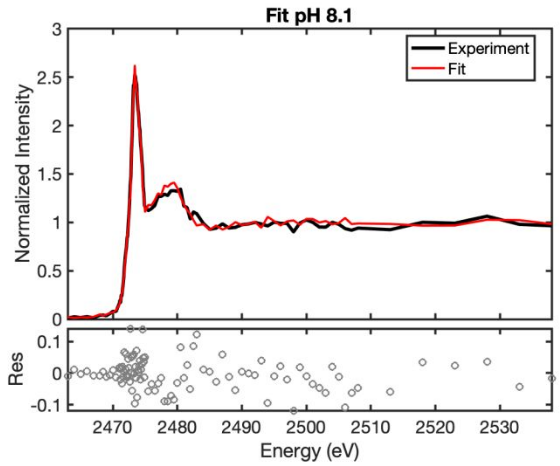

Figure S44. Fit of Cys S K-edge TFY XAS spectrum at pH 8.1 overlaid on experimental data with residuals plotted.

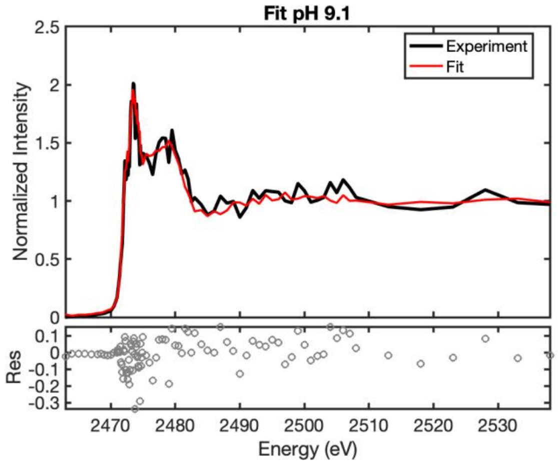

Figure S45. Fit of Cys S K-edge TFY XAS spectrum at pH 9.1 overlaid on experimental data with residuals plotted.

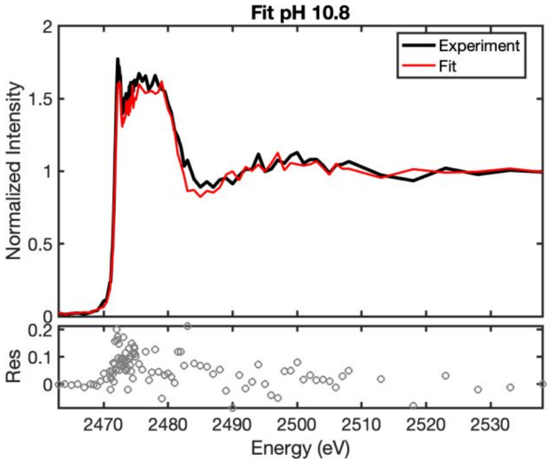

Figure S46. Fit of Cys S K-edge TFY XAS spectrum at pH 10.8 overlaid on experimental data with residuals plotted.

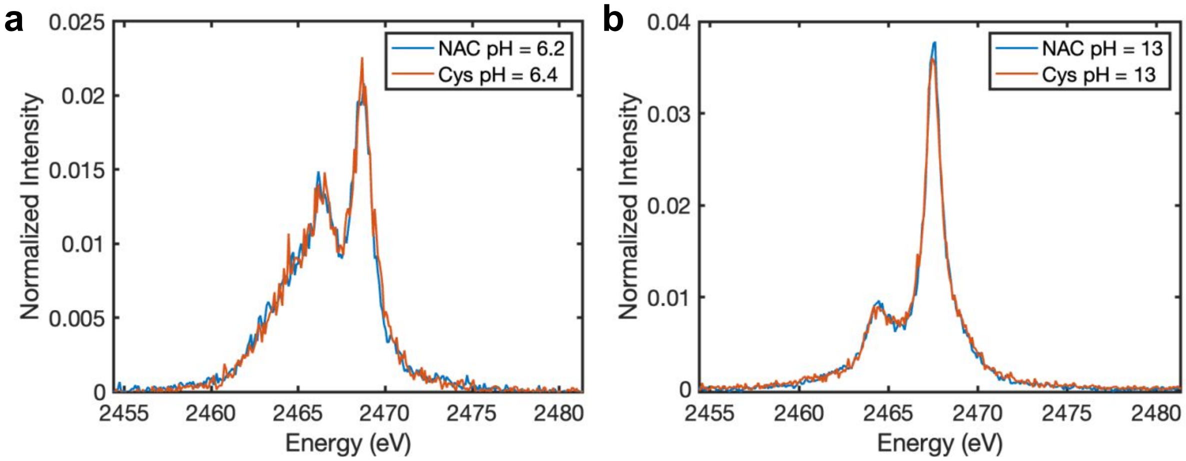

Figure S47. (a) Overlay of NAC and Cys S Kβ spectra at low pH (6.2 and 6.4 respectively) to compare the thiol spectra. (b) Overlay of NAC and Cys S Kβ spectra at high pH (13) to compare thiolate spectra.

DFT - HSNHAc

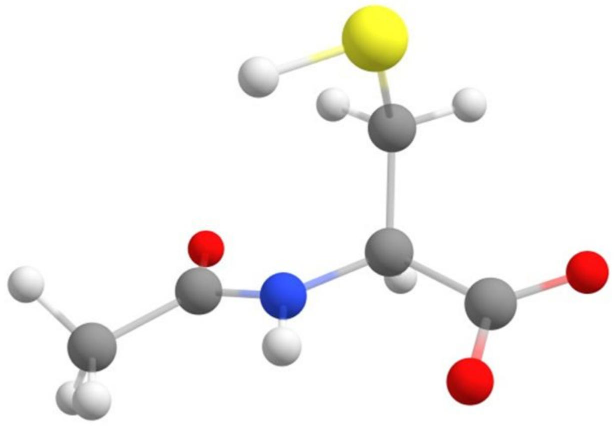

Figure S48. Optimized geometry of **HSNHAc**.

HSNHAc Optimized Geometry Coordinates:

S	-0.091878000	0.060368000	0.057953000
O	3.141230000	-1.929761000	0.328735000
O	1.413755000	-3.196140000	0.995775000
O	1.902364000	1.081112000	3.841935000
N	2.560402000	0.067908000	1.929597000
C	2.112922000	-2.158168000	1.010411000
C	1.644114000	-1.050202000	1.988754000
C	0.195695000	-0.639737000	1.720747000
C	2.651097000	1.029398000	2.858755000
C	3.721963000	2.069183000	2.632036000
H	1.667561000	-1.474688000	2.996551000
H	-0.139409000	0.073926000	2.467991000
H	-0.435844000	-1.523172000	1.768490000
H	0.692219000	1.139815000	0.193441000
H	3.243722000	0.002447000	1.189786000
H	3.243318000	3.034847000	2.463984000
H	4.362093000	1.839314000	1.782762000
H	4.330276000	2.153048000	3.532298000

HSNHAc VTC

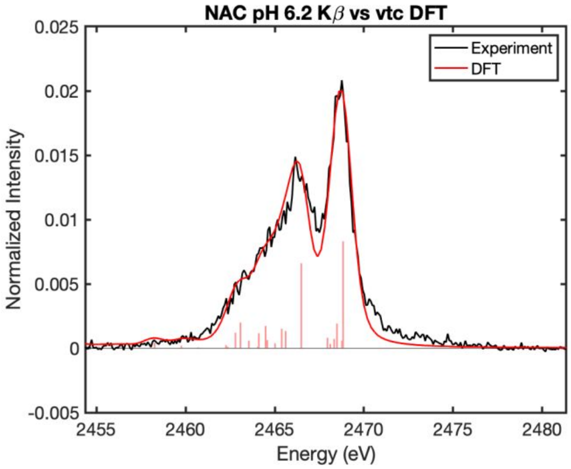

Figure S49. Overlay of experimental NAC S Kβ spectrum at pH 6.2 with the DFT-calculated S Kβ spectrum of the geometry optimized **HSNHAc** molecule. Voigtian linebroadening was applied to the stick spectrum with a Lorentzian linewidth of 0.522 eV (the S 1s core-hole lifetime) and a Gaussian full-width half-maximum of 1.1 eV. The DFT-calculated spectrum was shifted by 49.75 eV and scaled by 7.5e-6 to overlay with the area normalized experimental spectrum.

Table S4. The most intense Kβ transitions in the ground state DFT vtc calculation for **HSNHAc**.

Transition	Energy (eV)	Energy (eV) (Shifted 49.75)	Starting orbitals	Intensity
53	2413.321	2463.071	|27> -> |0>	0.00538
61	2414.721	2464.471	|31> -> |0>	0.00482
67	2415.643	2465.393	|34> -> |0>	0.00724
69	2415.847	2465.597	|35> -> |0>	0.00648
71	2416.749	2466.499	|36> -> |0>	0.02765
79	2418.738	2468.488	|40> -> |0>	0.00629
83	2419.079	2468.829	|42> -> |0>	0.03635

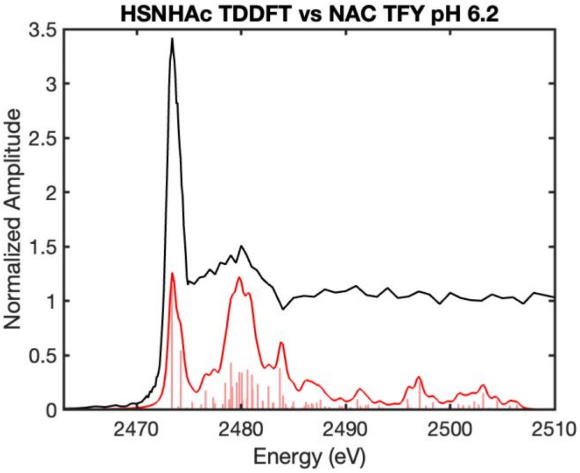

Figure S50. Overlay of experimental NAC TFY XAS spectrum at pH 6.2 with the TDDFT-calculated XAS spectrum of the geometry optimized **HSNHAc** molecule. Voigtian linebroadening was applied to the stick spectrum with a Lorentzian linewidth of 0.522 eV (the S 1s core-hole lifetime) and a Gaussian full-width half-maximum of 0.5 eV. The DFT-calculated spectrum was shifted by 49.5 eV and scaled by 1e-2 to overlay with the normalized experimental spectrum.

Table S5. TDDFT transitions for **HSNHAc** that contribute to the white line feature of the S K-edge XAS. The TDDFT transitions that contribute to the state are listed with their corresponding weights.

State	Energy (eV)	Energy (eV) (Shifted 49.5)	Starting orbitals	Contribution
2	2423.842	2473.342	|0> -> |43><br>|0> -> |44><br>|0> -> |45><br>|0> -> |46>	0.202583<br>0.177686<br>0.051608<br>0.052246
4	2424.687	2474.187	|0> -> |44><br>|0> -> |45><br>|0> -> |46><br>|0> -> |47>	0.115543<br>0.275528<br>0.010392<br>0.083746

Table S6. HSNHAc orbitals involved in calculated VTC and TDDFT transitions, a description of the nature of the orbital is given.

**|0>**	**|27>**
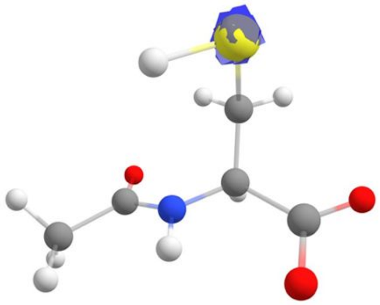	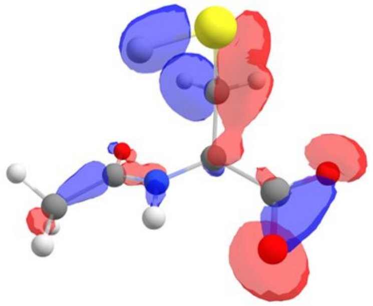
S 1s	S-H σ bonding
**|36>**	**|40>**
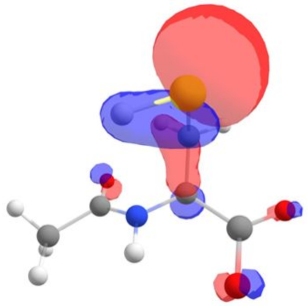	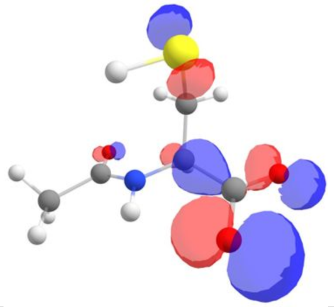
S-H/S-C σ bonding	S 3p non-bonding
**|43>**	**|44>**
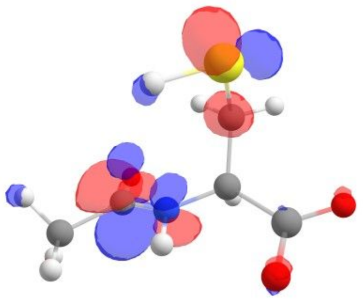	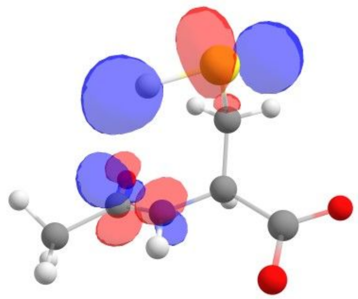
S-C/S-H σ* anti-bonding, acetyl π* anti-bonding	S-H σ* anti-bonding, acetyl π* anti-bonding

<table>
<tr><th>|45></th><th>|46></th></tr>
<tr><td>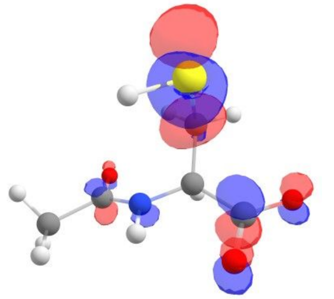</td><td>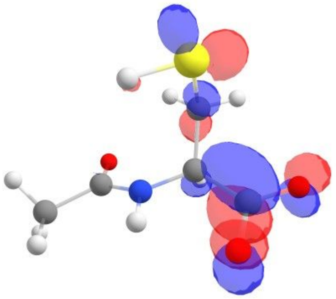</td></tr>
<tr><td>S-C σ^* anti-bonding, acetyl π^* anti-bonding</td><td>S-C/S-H σ^* anti-bonding, carboxylate π^* anti-bonding</td></tr>
<tr><th>|47></th><th></th></tr>
<tr><td>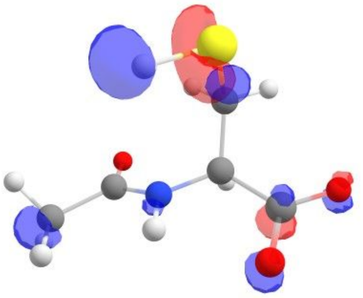</td><td></td></tr>
<tr><td>S-C/S-H σ^* anti-bonding</td><td></td></tr>
</table>

$^{-}$SNHAc

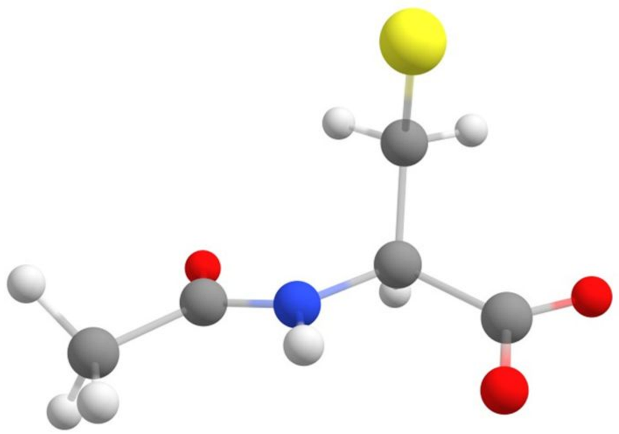

Figure S51. Optimized geometry of **$^{-}$SNHAc**.

$^{-}$SNHAc Optimized Geometry Coordinates:

S	-0.068665000	0.087471000	-0.008909000
O	3.137110000	-1.932078000	0.337408000
O	1.474958000	-3.239094000	1.076000000
O	1.967825000	1.039603000	3.904412000
N	2.558805000	0.060443000	1.948924000
C	2.124360000	-2.165747000	1.044538000
C	1.625804000	-1.047210000	1.986223000
C	0.183791000	-0.629373000	1.661671000
C	2.671373000	1.005257000	2.882875000
C	3.724063000	2.060759000	2.633265000
H	1.627807000	-1.457423000	3.000753000
H	-0.130222000	0.088386000	2.420023000
H	-0.433606000	-1.519828000	1.780461000
H	3.182378000	0.050862000	1.157465000
H	3.232082000	3.027351000	2.516236000
H	4.324304000	1.860249000	1.747805000
H	4.376232000	2.124872000	3.504250000

$^{-}$SNHAc VTC

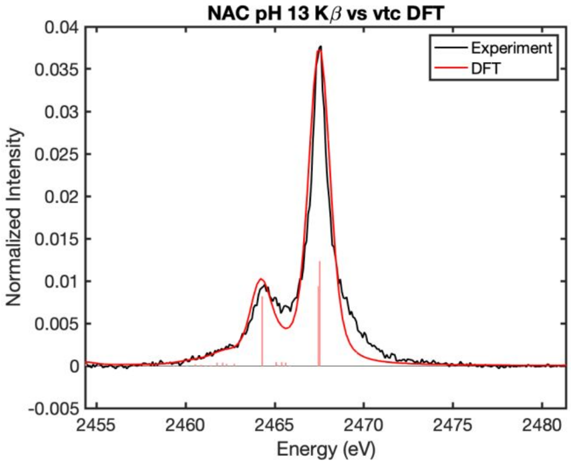

Figure S52. Overlay of experimental NAC S Kβ spectrum at pH 13 with the DFT-calculated S Kβ spectrum of the geometry optimized $^{-}$**SNHAc** molecule. Voigtian linebroadening was applied to the stick spectrum with a Lorentzian linewidth of 0.522 eV (the S 1s core-hole lifetime) and a Gaussian full-width half-maximum of 1.1 eV. The DFT-calculated spectrum was shifted by 49.3 eV and scaled by 7.5e-6 to overlay with the area normalized experimental spectrum.

Table S7. The most intense Kβ transitions in the ground state DFT vtc calculation for $^{-}$**SNHAc**.

Transition	Energy (eV)	Energy (eV) (Shifted 49.3)	Orbitals	Intensity
69	2414.985	2464.285	|35> -> |0>	0.00222
81	2418.149	2467.449	|41> -> |0>	0.04573
83	2418.220	2467.520	|42> -> |0>	0.04591

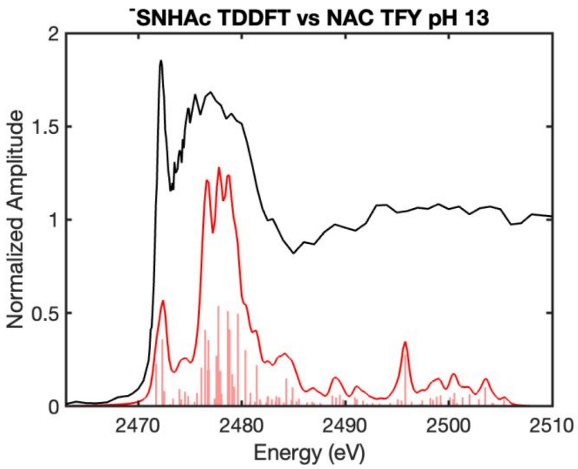

Figure S53. Overlay of experimental NAC TFY XAS spectrum at pH 13 with the TDDFT-calculated XAS spectrum of the geometry optimized $^{-}$**SNHAc** molecule. Voigtian linebroadening was applied to the stick spectrum with a Lorentzian linewidth of 0.522 eV (the S 1s core-hole lifetime) and a Gaussian full-width half-maximum of 0.5 eV. The DFT-calculated spectrum was shifted by 49.5 eV and scaled by 1e-2 to overlay with the normalized experimental spectrum.

Table S8. TDDFT transitions for $^{-}$**SNHAc** that contribute to the white line feature of the S K-edge XAS. The TDDFT transitions that contribute to the state are listed with their corresponding weights.

**Transition**	**Energy (eV)**	**Energy (eV) (Shifted 49.5)**	**Starting orbitals**	**Contribution**
2	2422.214	2463.071	|0> -> |43><br>|0> -> |45><br>|0> -> |47>	0.385200<br>0.030316<br>0.059953
4	2422.820	2463.071	|0> -> |43><br>|0> -> |44><br>|0> -> |45><br>|0> -> |46>	0.113365<br>0.019504<br>0.127090<br>0.023662

<table>
<tr><td></td><td></td><td></td><td>|0> -> |47></td><td>0.190643</td></tr>
</table>

Table S9. $^{-}$SNHAc orbitals involved in calculated VTC and TDDFT transitions, a description of the nature of the orbital is given.

<table>
<tr><th>|0></th><th>|35></th></tr>
<tr><td>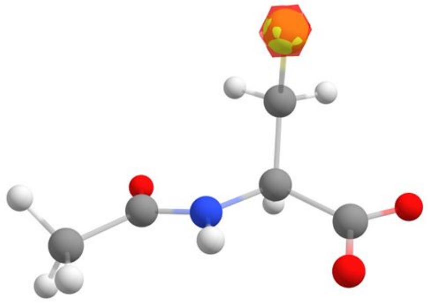</td><td>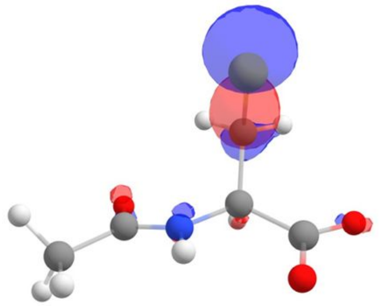</td></tr>
<tr><td>S 1s</td><td>S-C σ bonding</td></tr>
<tr><th>|41></th><th>|42></th></tr>
<tr><td>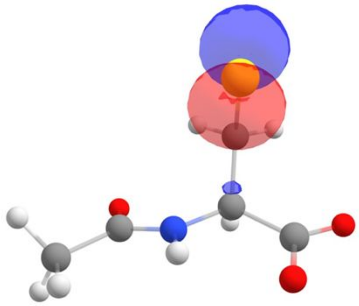</td><td>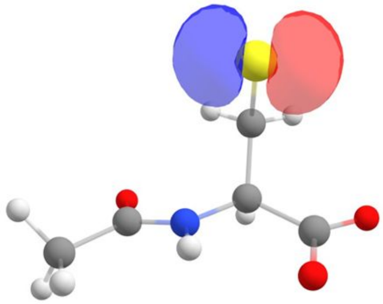</td></tr>
<tr><td>S 3p non-bonding</td><td>S 3p non-bonding</td></tr>
<tr><th>|43></th><th>|44></th></tr>
<tr><td>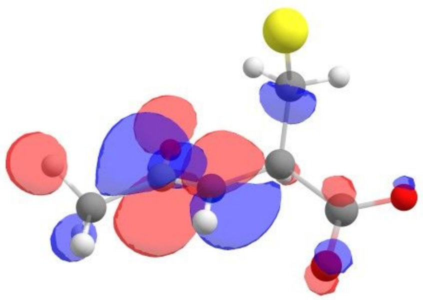</td><td>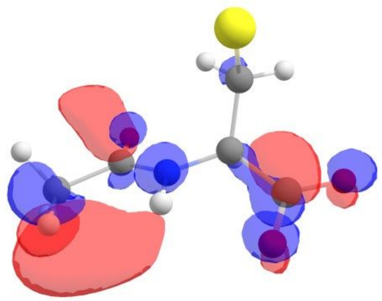</td></tr>
<tr><td>S 3p non-bonding</td><td>Acetyl, carboxylate π* anti-bonding</td></tr>
</table>

<table>
<tr><th>|45></th><th>|46></th></tr>
<tr><td>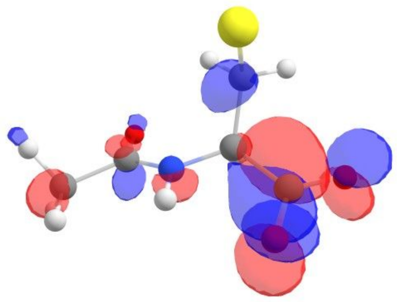</td><td>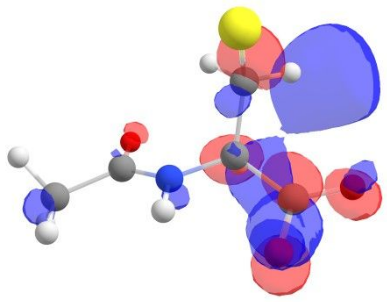</td></tr>
<tr><td>Carboxylate π* anti-bonding</td><td>S-C σ* anti-bonding, carboxylate π* anti-bonding</td></tr>
<tr><th>|47></th><th></th></tr>
<tr><td>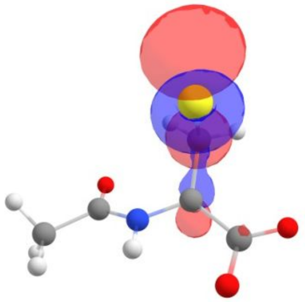</td><td></td></tr>
<tr><td>S-C σ* anti-bonding</td><td></td></tr>
</table>

Table S10. DFT calculated reduced Mulliken population (s+p) of **HSNHAc** and $^{-}$**SNHAc** compared to the experimental $K\alpha_1$ and $K\alpha_2$ peak positions of NAC at pH 6.2 and 13, respectively.

**pH**	**$K\alpha_1$ (eV)**	**$K\alpha_2$ (eV)**	**Mulliken Reduced Population (s+p)**
6.2	2308.374	2307.710	16.113494 (**HSNHAc**)
13.0	2308.326	2307.661	16.930538 ($^{-}$**SNHAc)**
Δ	0.048	0.049	0.8170

Cys – $HSNH_3^+$

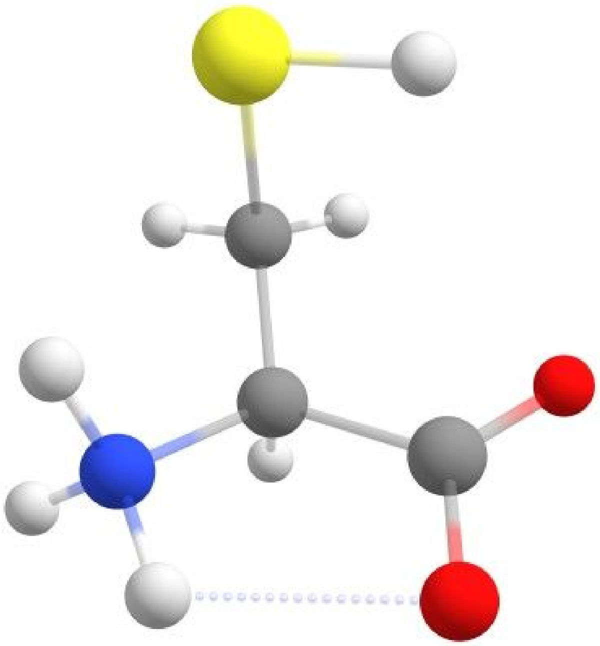

Figure S54. Optimized geometry of **$HSNH_3^+$**.

$HSNH_3^+$ Optimized Geometry Coordinates:

S	-1.958673000	-0.364753000	-0.543022000
C	-1.004739000	-0.060233000	0.984946000
C	0.382326000	0.503258000	0.734870000
C	1.330952000	-0.453837000	-0.041854000
N	0.323498000	1.787571000	-0.024009000
O	1.322407000	-1.643444000	0.321391000
O	2.024595000	0.084101000	-0.938961000
H	-1.178924000	-1.343940000	-1.026563000
H	-1.608110000	0.638237000	1.562846000
H	-0.911014000	-0.983213000	1.547044000
H	0.854076000	0.722088000	1.692988000
H	1.176849000	1.802731000	-0.601418000
H	-0.475829000	1.789261000	-0.662118000
H	0.272636000	2.609562000	0.571381000

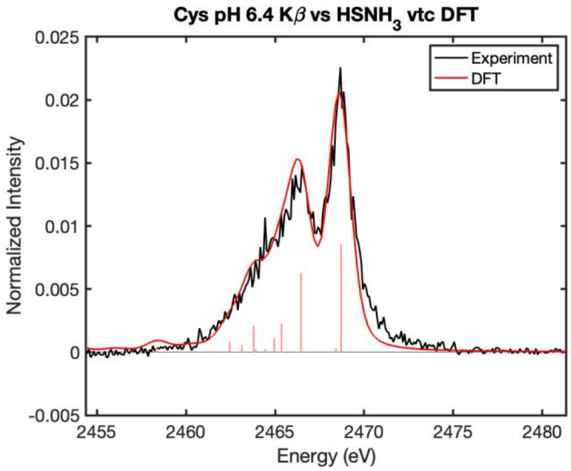

Figure S55. Overlay of experimental Cys S Kβ spectrum at pH 6.4 with the DFT-calculated S Kβ spectrum of the geometry optimized **$HSNH_3^+$** molecule. Voigtian linebroadening was applied to the stick spectrum with a Lorentzian linewidth of 0.522 eV (the S 1s core-hole lifetime) and a Gaussian full-width half-maximum of 1.1 eV. The DFT-calculated spectrum was shifted by 49.75 eV and scaled by 7.5e-6 to overlay with the area normalized experimental spectrum.

Table S11. The most intense Kβ transitions in the ground state DFT vtc calculation for **$HSNH_3^+$**.

**Transition**	**Energy (eV)**	**Energy (eV) (Shifted 49.75)**	**Orbitals**	**Intensity**
43	2414.051	2463.801	|22> -> |0>	0.01029
51	2415.615	2465.365	|26> -> |0>	0.01133
53	2416.707	2466.457	|27> -> |0>	0.03113

-

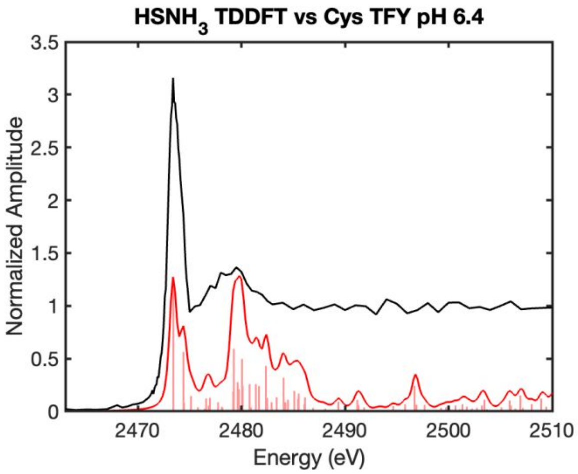

Figure S56. Overlay of experimental Cys TFY XAS spectrum at pH 6.4 with the TDDFT-calculated XAS spectrum of the geometry optimized **$HSNH_3^+$** molecule. Voigtian linebroadening was applied to the stick spectrum with a Lorentzian linewidth of 0.522 eV (the S 1s core-hole lifetime) and a Gaussian full-width half-maximum of 0.5 eV. The DFT-calculated spectrum was shifted by 49.5 eV and scaled by 1e-2 to overlay with the normalized experimental spectrum.

Table S12. TDDFT transitions for **$HSNH_3^+$** that contribute to the white line feature of the S K-edge XAS. The TDDFT transitions that contribute to the state are listed with their corresponding weights.

Transition	Energy (eV)	Energy (eV) (Shifted 49.5)	Starting orbitals	Contribution
2	2423.900	2473.400	|0> -> |32><br>|0> -> |33>	0.330287<br>0.147895
4	2424.922	2474.422	|0> -> |34><br>|0> -> |35>	0.324097<br>0.152970

Table S13. HSNH$_3^+$ orbitals involved in calculated VTC and TDDFT transitions, a description of the nature of the orbital is given.

**|0>**	**|22>**
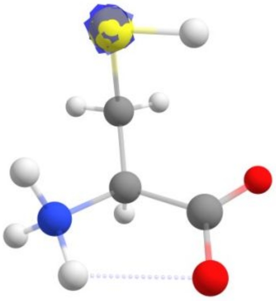	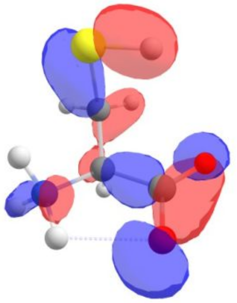
S 1s	S-C/S-H σ bonding
**|26>**	**|27>**
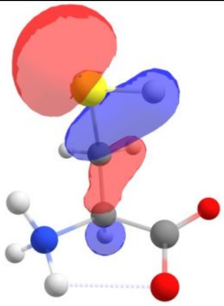	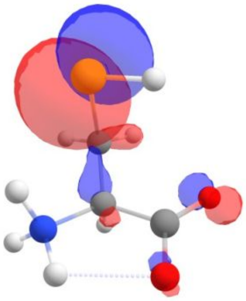
S-C/S-H σ bonding	S 3p non-bonding
**|32>**	**|33>**
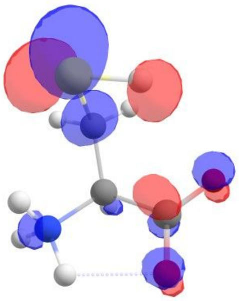	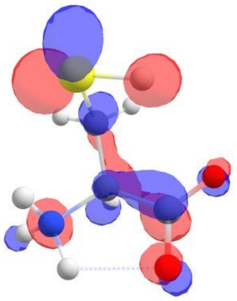
S-C/S-H σ* anti-bonding, carboxylate π* anti-bonding	S-C σ* anti-bonding, carboxylate π* anti-bonding

**|34>**	**|35>**
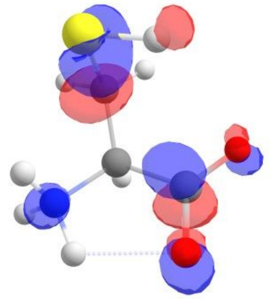	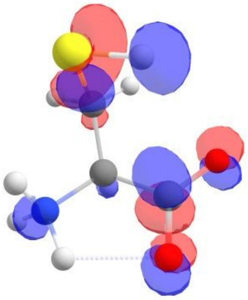
S-C σ* anti-bonding, carboxylate π* anti-bonding	S-C σ* anti-bonding, carboxylate π* anti-bonding

$^{-}$SNH$_2$

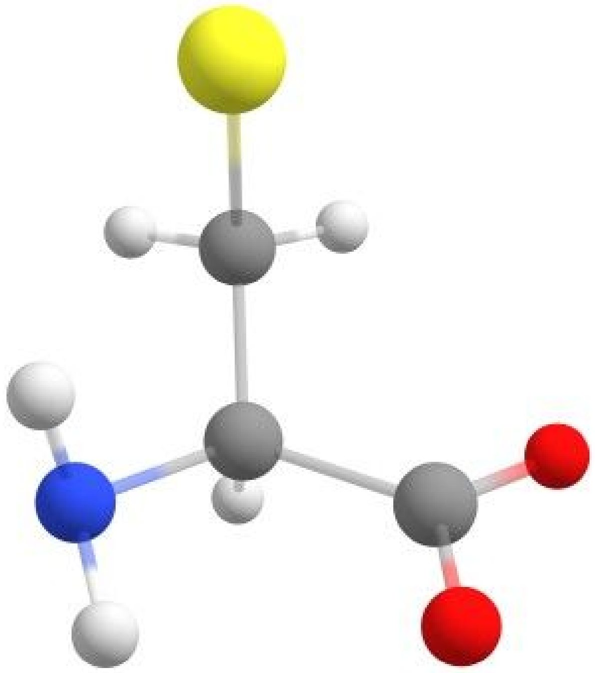

Figure S57. Optimized geometry of **$^{-}$SNH$_2$**.

-SNH$_2$ Optimized Geometry Coordinates:

S	-1.933894000	-0.244245000	-0.670798000
C	-0.991008000	-0.039431000	0.898159000
C	0.417027000	0.537619000	0.717427000
C	1.364606000	-0.467255000	0.016063000
N	0.345712000	1.854626000	0.078695000
O	1.464596000	-1.605554000	0.545108000
O	2.007439000	-0.075132000	-0.992238000
H	-1.547806000	0.624119000	1.564805000
H	-0.893599000	-1.001360000	1.397560000
H	0.838287000	0.662211000	1.724519000
H	-0.367006000	1.766297000	-0.645782000
H	1.213187000	1.989506000	-0.430427000

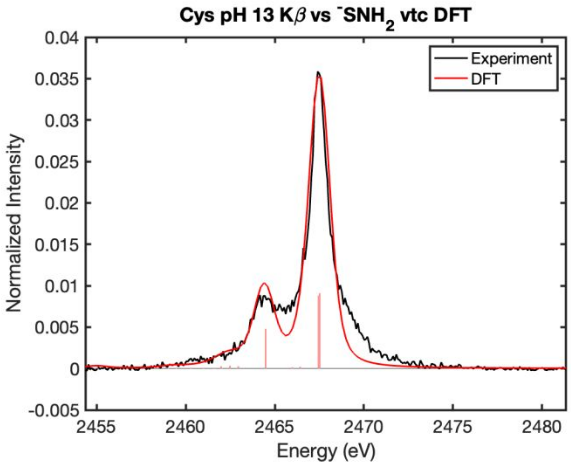

Figure S58. Overlay of experimental Cys S Kβ spectrum at pH 6.4 with the DFT-calculated S Kβ spectrum of the geometry optimized **$^-SNH_2$** molecule. Voigtian linebroadening was applied to the stick spectrum with a Lorentzian linewidth of 0.522 eV (the S 1s core-hole lifetime) and a Gaussian full-width half-maximum of 1.1 eV. The DFT-calculated spectrum was shifted by 49.3 eV and scaled by 7.4e-6 to overlay with the area normalized experimental spectrum.

Table S14. The most intense Kβ transitions in the ground state DFT vtc calculation for **$^-SNH_2$**.

**Transition**	**Energy (eV)**	**Energy (eV) (Shifted 49.3)**	**Orbitals**	**Intensity**
49	2415.189	2464.489	|25> -> |0>	0.02413
59	2418.143	2467.443	|30> -> |0>	0.04443
61	2418.225	2467.525	|31> -> |0>	0.04570

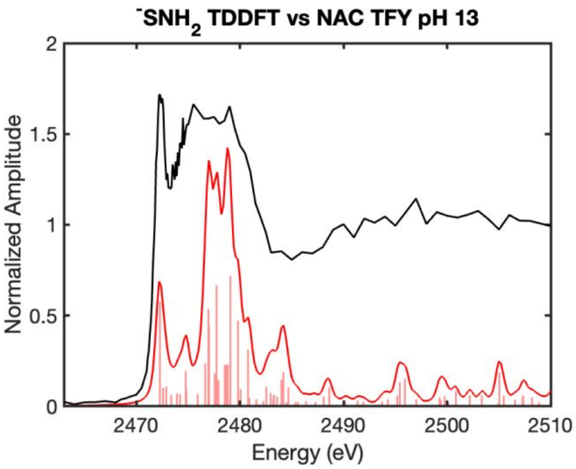

Figure S59. Overlay of experimental Cys TFY XAS spectrum at pH 13 with the TDDFT-calculated XAS spectrum of the geometry optimized $^-$**SNH$_2$** molecule. Voigtian linebroadening was applied to the stick spectrum with a Lorentzian linewidth of 0.522 eV (the S 1s core-hole lifetime) and a Gaussian full-width half-maximum of 0.5 eV. The DFT-calculated spectrum was shifted by 49.5 eV and scaled by 1e-2 to overlay with the normalized experimental spectrum.

Table S15. TDDFT transitions for $^-$**SNH$_2$** that contribute to the white line feature of the S K-edge XAS. The TDDFT transitions that contribute to the state are listed with their corresponding weights.

**Transition**	**Energy (eV)**	**Energy (eV) (Shifted 49.5)**	**Starting orbitals**	**Contribution**
2	2422.794	2472.294	|0> -> |32>	0.222025
			|0> -> |33>	0.048438
			|0> -> |34>	0.134801
			|0> -> |35>	0.065803
			|0> -> |36>	0.011148

Table S16. $^{-}$**SNH$_2$** orbitals involved in calculated VTC and TDDFT transitions, a description of the nature of the orbital is given.

<table>
<tr><th>|0></th><th>|25></th></tr>
<tr><td>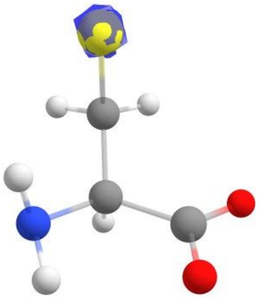</td><td>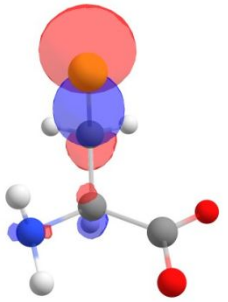</td></tr>
<tr><td>S 1s</td><td>S-C/S-H σ bonding</td></tr>
<tr><th>|30></th><th>|31></th></tr>
<tr><td>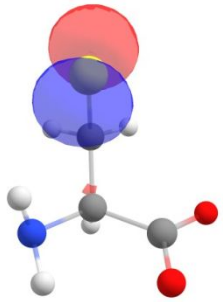</td><td>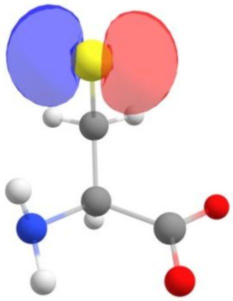</td></tr>
<tr><td>S 3p non-bonding</td><td>S 3p non-bonding</td></tr>
<tr><th>|32></th><th>|33></th></tr>
<tr><td>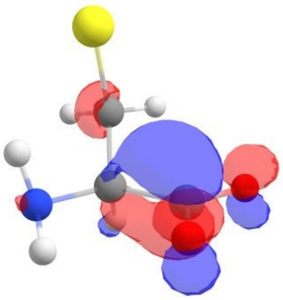</td><td>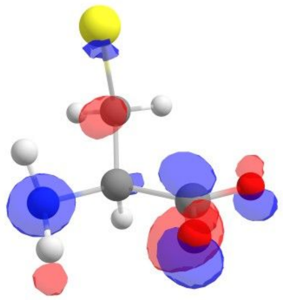</td></tr>
<tr><td>Carboxylate π* anti-bonding</td><td>Carboxylate π* anti-bonding</td></tr>
</table>

**|34>**	**|35>**
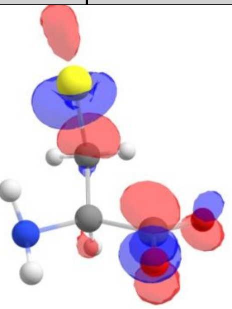	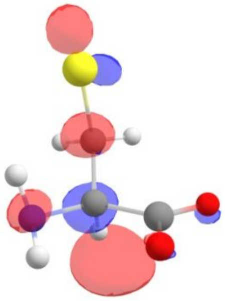
S-C σ* anti-bonding, carboxylate π* anti-bonding	S-C σ* anti-bonding
**|36>**	
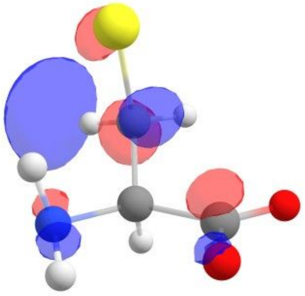	
S-C σ* anti-bonding	

$HSNH_2$

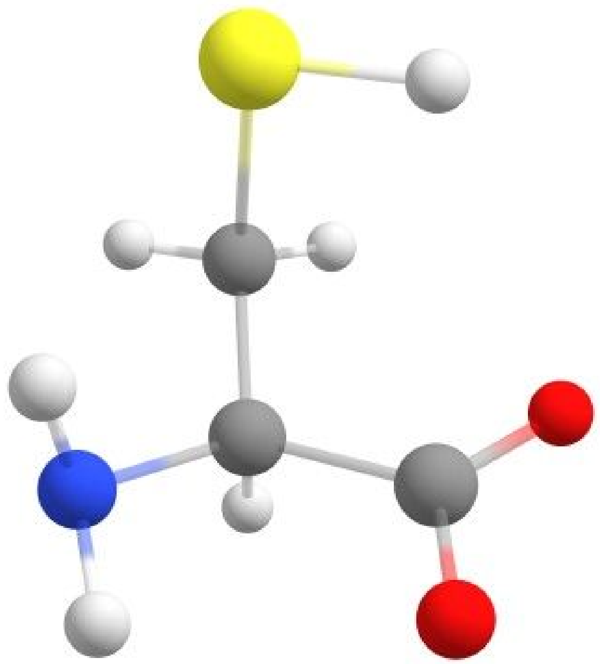

Figure S60. Optimized geometry of **$HSNH_2$**.

$HSNH_2$ Optimized Geometry Coordinates:

S	-1.958673000	-0.364753000	-0.543022000
C	-1.004739000	-0.060233000	0.984946000
C	0.382326000	0.503258000	0.734870000
C	1.330952000	-0.453837000	-0.041854000
N	0.323498000	1.787571000	-0.024009000
O	1.322407000	-1.643444000	0.321391000
O	2.024595000	0.084101000	-0.938961000
H	-1.178924000	-1.343940000	-1.026563000
H	-1.608110000	0.638237000	1.562846000
H	-0.911014000	-0.983213000	1.547044000
H	0.854076000	0.722088000	1.692988000
H	1.176849000	1.802731000	-0.601418000
H	-0.475829000	1.789261000	-0.662118000
H	0.272636000	2.609562000	0.571381000

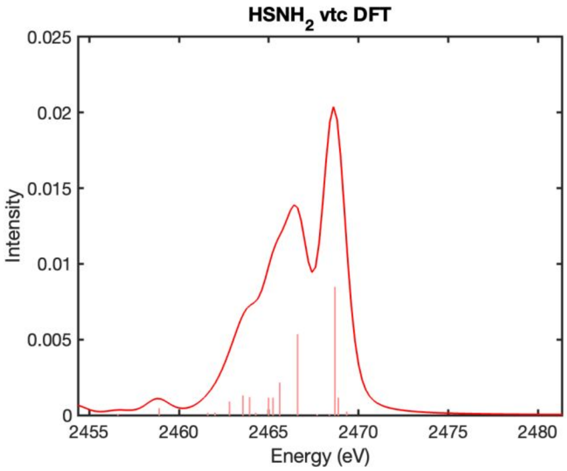

Figure S61. The DFT-calculated S Kβ spectrum of the geometry optimized **$HSNH_2$** molecule. Voigtian linebroadening was applied to the stick spectrum with a Lorentzian linewidth of 0.522 eV (the S 1s core-hole lifetime) and a Gaussian full-width half-maximum of 1.1 eV. The DFT-calculated spectrum was shifted by 49.75 eV and scaled by 7.5e-6.

Table S17. The most intense Kβ transitions in the ground state DFT vtc calculation for **$HSNH_3$**.

Transition	Energy (eV)	Energy (eV) (Shifted 49.75)	Orbitals	Intensity
39	2413.790	2463.540	|20> -> |0>	0.00644
49	2415.850	2465.600	|25> -> |0>	0.01066
51	2416.849	2466.599	|26> -> |0>	0.02661

-

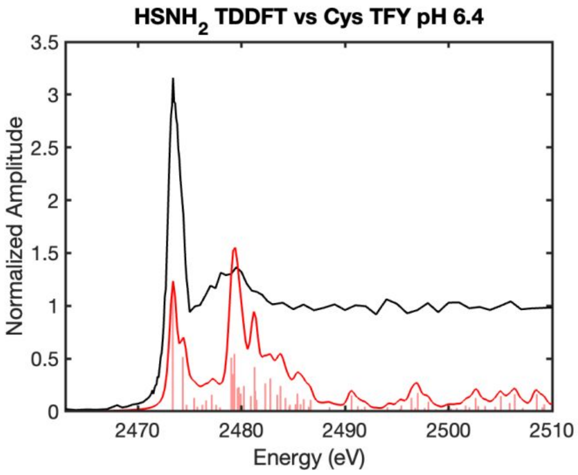

Figure S62. Overlay of experimental Cys TFY XAS spectrum at pH 6.4 with the TDDFT-calculated XAS spectrum of the geometry optimized **$HSNH_2$** molecule. Voigtian linebroadening was applied to the stick spectrum with a Lorentzian linewidth of 0.522 eV (the S 1s core-hole lifetime) and a Gaussian full-width half-maximum of 0.5 eV. The DFT-calculated spectrum was shifted by 49.5 eV and scaled by 1e-2 to overlay with the normalized experimental spectrum.

Table S18. TDDFT transitions for **$HSNH_2$** that contribute to the white line feature of the S K-edge XAS. The TDDFT transitions that contribute to the state are listed with their corresponding weights.

Transition	Energy (eV)	Energy (eV) (Shifted 49.5)	Starting orbitals	Contribution
2	2423.844	2473.344	|0> -> |32><br>|0> -> |34>	0.426190<br>0.050705
4	2424.818	2474.318	|0> -> |33><br>|0> -> |34><br>|0> -> |35>	0.307016<br>0.120135<br>0.057799

Table S19. $HSNH_2$ orbitals involved in calculated VTC and TDDFT transitions, a description of the nature of the orbital is given.

**|0>**	**|25>**
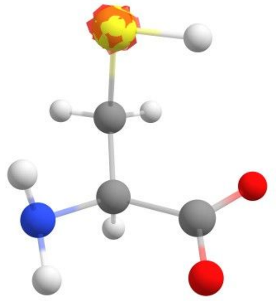	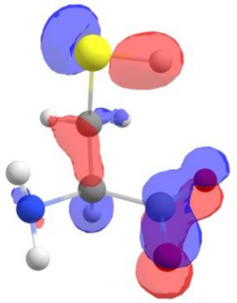
S 1s	S-H σ bonding
**|26>**	**|29>**
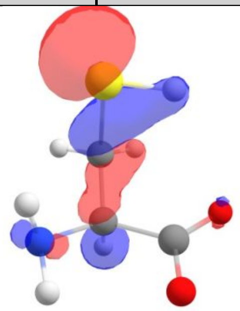	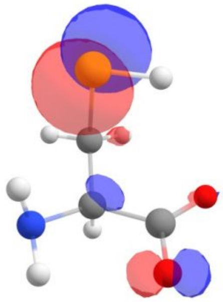
S-C/S-H σ bonding	S 3p non-bonding
**|32>**	**|33>**
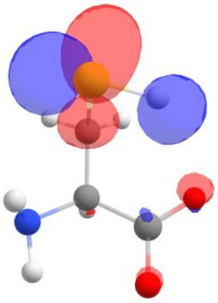	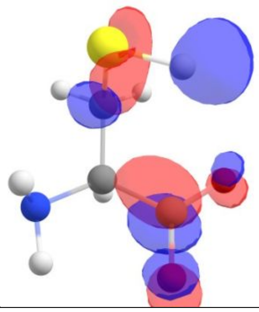
S-C/S-H σ* anti-bonding	S-C/S-H σ* anti-bonding, carboxylate π* anti-bonding

**$\vert 34\rangle$**	**$\vert 35\rangle$**
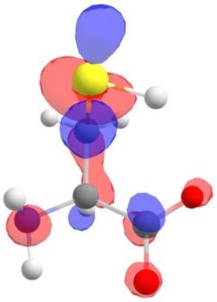	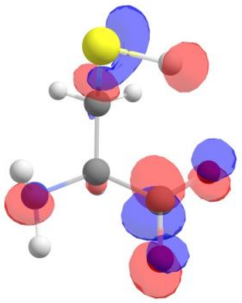
S-C σ* anti-bonding, carboxylate π* anti-bonding	S-C/S-H σ* anti-bonding, carboxylate π* anti-bonding

$^{-}$SNH$_3^+$

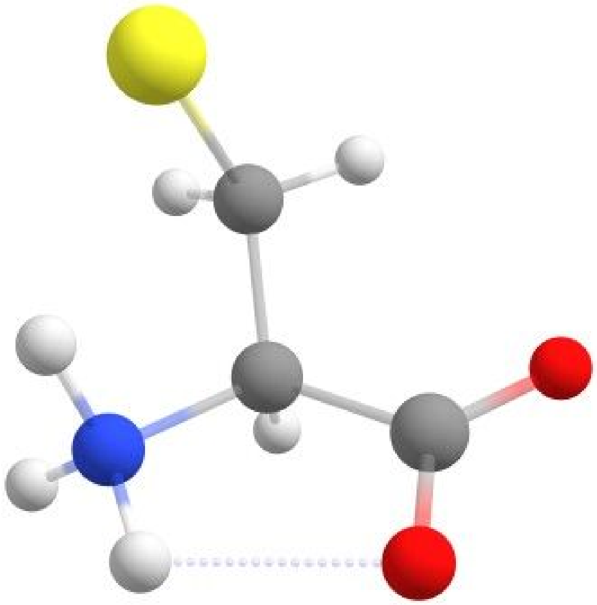

Figure S63. Optimized geometry of **$^{-}$SNH$_3^+$**.

$^{-}$SNH$_3^+$ Optimized Geometry Coordinates:

S	-1.984487000	-0.048768000	-0.598638000
C	-1.009370000	-0.055093000	0.957683000
C	0.398485000	0.512735000	0.756714000
C	1.373623000	-0.448154000	0.030547000
N	0.312431000	1.764226000	-0.048894000
O	1.466151000	-1.599484000	0.500843000
O	2.013586000	0.034770000	-0.939529000
H	-1.524426000	0.533419000	1.718807000
H	-0.891770000	-1.065541000	1.341660000
H	0.846655000	0.766698000	1.717892000
H	1.137233000	1.782623000	-0.658647000
H	-0.540575000	1.647424000	-0.641017000
H	0.244483000	2.610144000	0.507869000

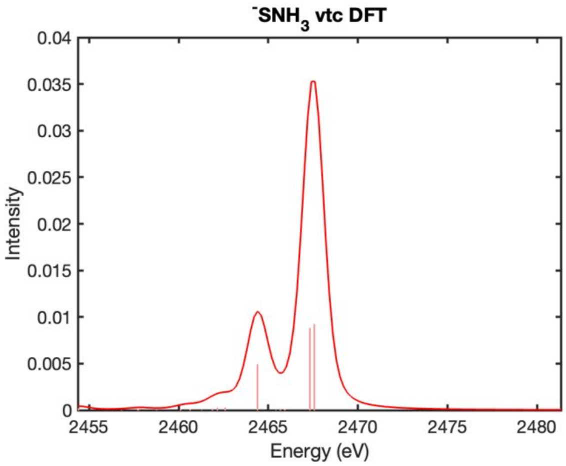

Figure S64. The DFT-calculated S Kβ spectrum of the geometry optimized $^{-}$**SNH$_3^+$** molecule. Voigtian linebroadening was applied to the stick spectrum with a Lorentzian linewidth of 0.522 eV (the S 1s core-hole lifetime) and a Gaussian full-width half-maximum of 1.1 eV. The DFT-calculated spectrum was shifted by 49.3 eV and scaled by 7.4e-6.

Table S20. The most intense Kβ transitions in the ground state DFT vtc calculation for $^{-}$**SNH$_3^+$**.

**Transition**	**Energy (eV)**	**Energy (eV) (Shifted 42.3)**	**Orbitals**	**Intensity**
51	2415.082	2457.382	|26> -> |0>	0.02491
59	2418.031	2460.331	|30> -> |0>	0.04433
61	2418.226	2460.526	|31> -> |0>	0.04637

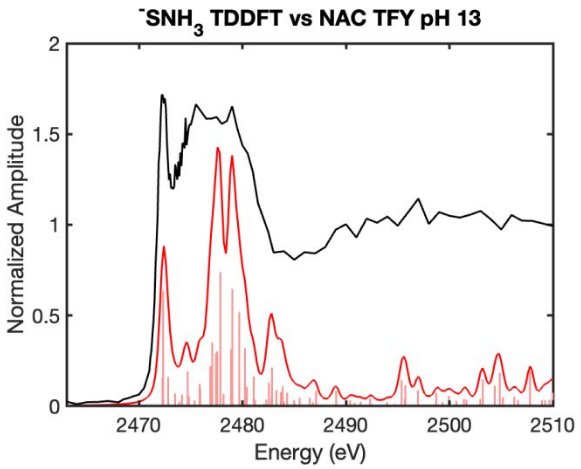

Figure S65. Overlay of experimental Cys TFY XAS spectrum at pH 13 with the TDDFT-calculated XAS spectrum of the geometry optimized **$^{-}$SNH$_3^+$** molecule. Voigtian linebroadening was applied to the stick spectrum with a Lorentzian linewidth of 0.522 eV (the S 1s core-hole lifetime) and a Gaussian full-width half-maximum of 0.5 eV. The DFT-calculated spectrum was shifted by 49.5 eV and scaled by 1e-2 to overlay with the normalized experimental spectrum.

Table S21. TDDFT transitions for **$^{-}$SNH$_3^+$** that contribute to the white line feature of the S K-edge XAS. The TDDFT transitions that contribute to the state are listed with their corresponding weights.

Transition	Energy (eV)	Energy (eV) (Shifted 49.5)	Starting orbitals	Contribution
2	2422.824	2472.324	|0> -> |32><br>|0> -> |33><br>|0> -> |34><br>|0> -> |35>	0.153664<br>0.089314<br>0.172377<br>0.055796

Table S22. $^{-}$**SNH$_3$$^{+}$** orbitals involved in calculated VTC and TDDFT transitions, a description of the nature of the orbital is given.

<table>
<tr><th>|0></th><th>|26></th></tr>
<tr><td>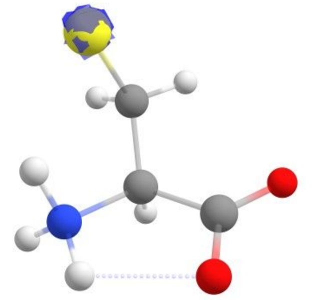</td><td>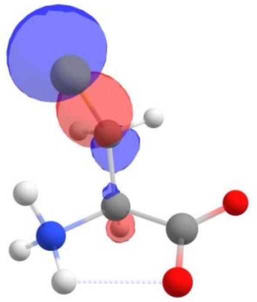</td></tr>
<tr><td>S 1s</td><td>S-C σ bonding</td></tr>
<tr><th>|30></th><th>|31></th></tr>
<tr><td>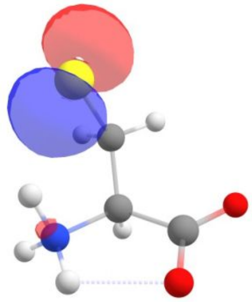</td><td>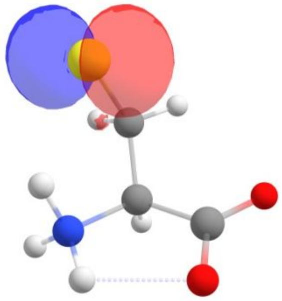</td></tr>
<tr><td>S 3p non-bonding</td><td>S 3p non-bonding</td></tr>
<tr><th>|32></th><th>|33></th></tr>
<tr><td>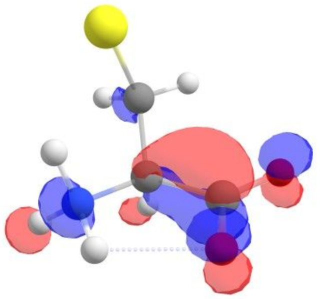</td><td>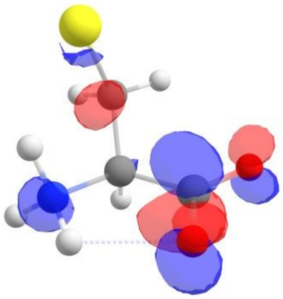</td></tr>
<tr><td>Carboxylate π* anti-bonding</td><td>Carboxylate π* anti-bonding</td></tr>
</table>

|34>	|35>
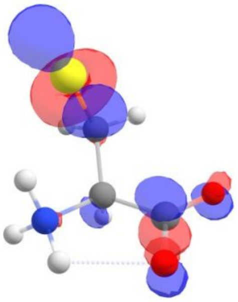	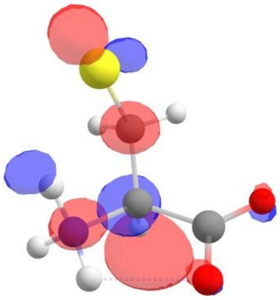
S-C σ* anti-bonding, carboxylate π* anti-bonding	S-C σ* anti-bonding

Table S23. DFT calculated Mulliken reduced population densities for **$HSNH_3^+$**, **$^-SNH_2$**, **$HSNH_2$**, and **$^-SNH_3^+$**.

**$HSNH_3^+$**		**$^-SNH_2$**		**$HSNH_2$**		**$^-SNH_3^+$**	
s	5.288376	s	5.521746	s	5.290611	s	5.512628
pz	3.180452	pz	3.340326	pz	3.236442	pz	3.316406
px	3.474077	px	3.647029	px	3.488894	px	3.609882
py	3.411790	py	3.827536	py	3.384296	py	3.779258
Total (s + p)	15.354695	Total (s + p)	16.336637	Total (s + p)	15.400243	Total (s + p)	16.218174

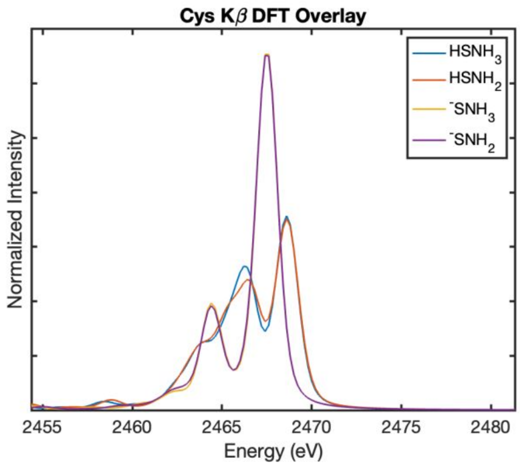

Figure S66. A comparison of the DFT-calculated S Kβ spectra of the geometry optimized **$HSNH_3$**, **$HSNH_2$**, $^-$**SNH_3**, and $^-$**SNH_2** molecules.

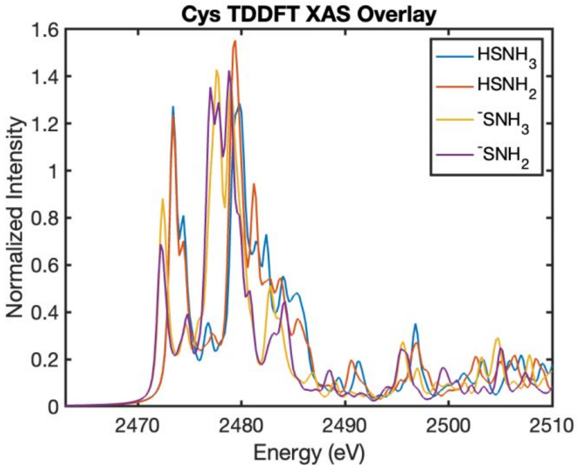

Figure S67. A comparison of the TDDFT-calculated S K-edge TFY XAS spectra of the geometry optimized **$HSNH_3$**, **$HSNH_2$**, $^-$**SNH_3**, and $^-$**SNH_2** molecules.

References

(1) Abraham, B.; Nowak, S.; Weninger, C.; Armenta, R.; Defever, J.; Day, D.; Carini, G.; Nakahara, K.; Gallo, A.; Nelson, S.; Nordlund, D.; Kroll, T.; Hunter, M. S.; van Driel, T.; Zhu, D.; Weng, T.-C.; Alonso-Mori, R.; Sokaras, D. A High-Throughput Energy-Dispersive Tender X-Ray Spectrometer for Shot-to-Shot Sulfur Measurements. *J. Synchrotron Radiat.* **2019**, *26* (3), 629–634. https://doi.org/10.1107/S1600577519002431.

(2) Alonso Mori, R.; Paris, E.; Giuli, G.; Eeckhout, S. G.; Kavčič, M.; Žitnik, M.; Bučar, K.; Pettersson, L. G. M.; Glatzel, P. Electronic Structure of Sulfur Studied by X-Ray Absorption and Emission Spectroscopy. *Anal. Chem.* **2009**, *81* (15), 6516–6525. https://doi.org/10.1021/ac900970z.

(3) Benesch, R. E.; Benesch, R. The Acid Strength of the -SH Group in Cysteine and Related Compounds. *J. Am. Chem. Soc.* **1955**, *77* (22), 5877–5881. https://doi.org/10.1021/ja01627a030.

(4) Bodner, G. M. Assigning the pKa's of Polyprotic Acids. *J. Chem. Educ.* **1986**, *63* (3), 246. https://doi.org/10.1021/ed063p246.

(5) Neese, F. The ORCA Program System. *WIREs Comput. Mol. Sci.* **2012**, *2* (1), 73–78. https://doi.org/10.1002/wcms.81.

(6) Weigend, F.; Ahlrichs, R. Balanced Basis Sets of Split Valence, Triple Zeta Valence and Quadruple Zeta Valence Quality for H to Rn: Design and Assessment of Accuracy. *Phys. Chem. Chem. Phys.* **2005**, *7* (18), 3297–3305. https://doi.org/10.1039/B508541A.

(7) Campbell, J. L.; Papp, T. WIDTHS OF THE ATOMIC *K–N*7 LEVELS. *At. Data Nucl. Data Tables* **2001**, *77* (1), 1–56. https://doi.org/10.1006/adnd.2000.0848.
