# Supplementary material for: Determination of Thiol Protonation States by Sulfur X-ray Spectroscopy in Biological Systems
Source: J Phys Chem Lett. 2025 Feb 27;16(9):2401–8. doi: 10.1021/acs.jpclett.4c03247 (PMC11892467; doi:10.1021/acs.jpclett.4c03247)
Supplement: Supplementary file 2 — jz4c03247_si_002.pdf [file jz4c03247_si_002.pdf]

jz-2024-03247g.R1

Name: Peer Review Information for "Determination of Thiol Protonation States by Sulfur X-ray Spectroscopy in Biological Systems"

First Round of Reviewer Comments

Reviewer: 1

Comments to the Author
This is an interesting manuscript on the spectral sensitivity of S vtc XES, Ka XES and XANES in cysteine. From what I understand, the main message is that the spectra change significantly with protonation in the first coordination sphere of S but not or barely of the NH2 group. This is not unexpected and I do not understand the need for SVD and a lengthy discussion on ranks. What would be much more interesting is a discussion on what is needed to observe spectral differences between NAC and cysteine and the protonation of the NH2 group. What are the current experimental limiations and how can they be overcome? I therefore recommend to considerably reduce (or even completely remove) the SVD analysis, as I do not see the point, and focus the discussion on how subtle spectral changes could be ovserved.

Reviewer: 2

Comments to the Author
This paper presents new and interesting analysis of sulfur emission spectra involving thiol/thiolate groups. The comparison of K-alpha and K-beta spectra and the ability to detect differences in bonding are exciting. The manuscript overall is clear, well-written, and informative. However, the results and conclusion are somewhat tentative, particularly for the spectral analysis of cysteine. While the abstract promises the ability to probe the covalency of sulfur ligands, the concluding paragraph does not make a clear statement about exactly how the spectral analysis quantified this for both NAC and cysteine, and why it is broadly important. There are a number of vague statements about "sensitivity" of spectra and a "foundation" for future studies, but I could not grasp the take-away message that I could apply to my thinking about thiol and cysteine bonding.

The key figure for cysteine as a function of pH is Fig. 5, which presents an interesting set of spectra but no analysis of it. The associated text is long-winded about spectral fitting and statistical significance, but all of the actual data analysis is buried in supporting information, which is lengthy. I suggest revision of the latter half of the paper to make clear how these spectra were analyzed, and a succinct discussion of how the analysis supports the main conclusions of the paper within some estimate of uncertainty. The main text should present a convincing analysis with essential supporting details in the SI. The concluding paragraph needs to state in more definitive language the impact of the study results for the general reader.

Author's Response to Peer Review Comments:

Reviewer(s)' Comments to Author:

Reviewer: 1

Recommendation: This paper may be publishable, but major revision is needed; I would like to be invited to review any future revision.

Comments:
This is an interesting manuscript on the spectral sensitivity of S vtc XES, Ka XES and XANES in cysteine. From what I understand, the main message is that the spectra change significantly with protonation in the first coordination sphere of S but not or barely of the NH2 group. This is not unexpected and I do not understand the need for SVD and a lengthy discussion on ranks. What would be much more interesting is a discussion on what is needed to observe spectral differences between NAC and cysteine and the protonation of the NH2 group. What are the current experimental limiations and how can they be overcome? I therefore recommend to considerably reduce (or even completely remove) the SVD analysis, as I do not see the point, and focus the discussion on how subtle spectral changes could be ovserved.

We thank the reviewer for the insightful comments. We have addressed the points below.

"I therefore recommend to considerably reduce (or even completely remove) the SVD analysis"

- **Here, we have removed the bulk of this SVD discussion and replaced it with one statement in summary on pg. 15:**
    - **"SVD of the Cys S Kβ, Kα, and TFY XAS data matrices all show two major components, which is consistent with there being two unique spectra contributing to the data representing thiol and thiolate, irrespective of amine protonation."**

"What would be much more interesting is a discussion on what is needed to observe spectral differences between NAC and cysteine and the protonation of the NH2 group"

- **We have included three paragraphs of discussion after the Cys results (beginning pg. 15) on the lack of difference observed between NAC and Cys at low and high pH, on what kinds of chemical changes will be most apparent via S XES, and on current limitations for high-resolution, high signal-to-noise tender XES**

"These results provide a foundation for studying Cys chemistry in proteins using S X-ray spectroscopy and point to two direct applications: (1) to assess Cys thiol pK_a in protein systems where other techniques are ambiguous and (2) to detect bonding changes at Cys thiolates associated with catalytic activity in cysteine dependent enzymes. In pursuit of the second aim, it is valuable considering to which covalent changes these techniques will be most sensitive. From our results, we find S Kβ XES to be a good diagnostic tool to distinguish thiol and thiolate protonation states in NAC and Cys, comparable to S K-edge XAS. However, these spectroscopies are much less sensitive to distal covalent changes in cysteine, including amine protonation (in Cys) and acetylation (comparing NAC and Cys). Qureshi et al. reported similarly little sensitivity in the S Kβ XES in aliphatic thiols and sulfides outside of ring strain effects in cyclic systems. In comparison, Qureshi et al. found S Kβ XES to be more sensitive to ring substituents in a

series of thiophenes, where the S 3p orbitals are involved in a delocalized aromatic system.[34]

As expected, the S Kβ XES is most informative when differentiating a change in S bonding that directly impacts the distribution of S 3p electrons. For Cys, this sensitivity could include S protonation state change, intermediate formation following nucleophilic attack of a Cys thiolate, or potentially changes in the hydrogen bonding network around S in a protein. Cysteine dependent enzymes like isocyanide hydratase (ICH) are good examples where these techniques could elucidate Cys chemistry critical to protein function. ICH catalyzes the hydration of isocyanide substrates to formamides via a thioimidate formed with the active site Cys thiolate, whose reactivity is modulated by a local hydrogen bonding network.[43] S X-ray spectroscopy could serve as an appropriate probe for studying the chemistry and bonding occurring at the active site Cys as the enzyme proceeds through turnover.

Any such spectral changes may be slight, particularly in time-resolved measurements where only a fraction of the species may be converted at any given time. Even for Cys, where small spectral differences in the lower energy range of the S Kβ XES were predicted by DFT for $HSNH_3^+$ and $HSNH_2$, such differences were not resolved experimentally. High resolution and high signal-to-noise S Kβ XES is thus a priority for studying S chemistry in proteins but presents a current limitation. For our present study, NAC and Cys samples were prepared at 50 mM concentrations, which is well above the solubility limits for many proteins. However, the concentration constraints can be overcome by combining these measurements with serial crystallography experiments on crystalline protein samples and technological developments such as a new multielement von Hamos type tender X-ray spectrometer that is being prepared for use at the Linac Coherent Light Source and Stanford Synchrotron Radiation Lightsource to enable study of lower concentration samples with high signal-to-noise."

Additional Questions:
Urgency: High

Significance: High

Novelty: High

Scholarly Presentation: High

Is the paper likely to interest a substantial number of physical chemists, not just specialists working in the authors' area of research?: Yes

Reviewer: 2

Recommendation: This paper is probably publishable, but major revision is needed; I do not need to see future revisions.

Comments:
This paper presents new and interesting analysis of sulfur emission spectra involving thiol/thiolate groups. The comparison of K-alpha and K-beta spectra and the ability to detect differences in bonding are exciting. The manuscript overall is clear, well-written, and informative. However, the results and conclusion are somewhat tentative, particularly for the spectral analysis of cysteine. While the abstract promises the ability to probe the covalency of sulfur ligands, the concluding paragraph does not make a clear statement about exactly how the spectral analysis quantified this for both NAC and cysteine, and why it is broadly important. There are a number of vague statements about "sensitivity" of spectra and a "foundation" for future studies, but I could not grasp the take-away message that I could apply to my thinking about thiol and cysteine bonding.

The key figure for cysteine as a function of pH is Fig. 5, which presents an interesting set of spectra but no analysis of it. The associated text is long-winded about spectral fitting and statistical significance, but all of the actual data analysis is buried in supporting information, which is lengthy. I suggest revision of the latter half of the paper to make clear how these spectra were analyzed, and a succinct discussion of how the analysis supports the main conclusions of the paper within some estimate of uncertainty. The main text should present a convincing analysis with essential supporting details in the SI. The concluding paragraph needs to state in more definitive language the impact of the study results for the general reader.

We thank the reviewer for the recommendations, and we have taken the following steps to improve the paper by clarifying the discussion and conclusions.

"There are a number of vague statements about "sensitivity" of spectra and a "foundation" for future studies, but I could not grasp the take-away message that I could apply to my thinking about thiol and cysteine bonding."

- **We have tried to be more specific in various parts of the paper when discussing the "sensitivity" of the techniques. Here, our priority is to determine how the spectroscopy distinguishes chemical changes at the Cys sulfur. The utility of the techniques lies in our ability to determine chemical speciation and track chemical changes in Cys. We have adjusted the language to be more precise about that.**

"The key figure for cysteine as a function of pH is Fig. 5, which presents an interesting set of spectra but no analysis of it. The associated text is long-winded about spectral fitting and statistical significance, but all of the actual data analysis is buried in supporting information, which is lengthy. I suggest revision of the latter half of the paper to make clear how these spectra were analyzed, and a succinct discussion of how the analysis supports the main conclusions of the paper within some estimate of uncertainty."

- **We have removed the bulk of the discussion on SVD and spectral fitting and added more detail to the Cys results and analysis. Our principal analysis of the S Kβ XES is via DFT simulation of the valence-to-core transitions, which illuminates the origin of the differences observed between thiol and thiolate. The DFT also suggests why**

there is little difference NAC and Cys when comparing thiol to thiol and thiolate to thiolate as the S 3p electrons are little affected by N acetylation.

- **The discussion includes three additional paragraphs that the utility and limitations of the techniques in the context of our findings (also in Reviewer 1's comments).**
- **We have also rewritten the conclusion section (pg. 17) to better impress the take home message of the paper. Our results given insight into the how chemical changes at the cysteine sulfur can be observed in the S XES and XAS. The broader importance is the ability to apply these findings to protein systems where Cys pK_a determination can be ambiguous and to be able to study bonding changes at Cys S in cysteine dependent enzymes.**

"In conclusion, we demonstrate the sensitivity of S K-line X-ray emission to pH-dependent chemical changes in NAC and Cys. The thiol and thiolate protonation states of NAC and Cys are well distinguished by S Kβ XES and compares favorably with S K-edge XAS. DFT simulation of the VTC transitions provides good agreement with the measured Kβ spectra, allowing us to analyze the valence orbital contributions to these transitions. The distribution of S 3p orbitals between hybrid bonding orbitals and non-bonding orbitals is key for understanding the Kβ differences observed between thiol and thiolate. This also suggests why little difference is observed comparing NAC and Cys data, as covalent changes at the amine do not significantly alter the S 3p electrons. S Kα XES exhibits a small shift (~0.05 eV) moving from thiol to thiolate that is consistent with increased electron density in the thiolate S 3p orbitals compared to the thiol. Although the data can distinguish this change in the S Kα data, it serves as the least accurate of the three techniques for determining speciation at intermediate pH. Understanding sulfur ionization and bonding is an important challenge common to small molecule and macromolecular samples. We thus show that S K-line XES, especially the Kβ line, can be a potent tool to assess physiologically relevant chemical changes at the Cys sulfur. This analysis can be directly applied for assessing Cys S ionization and pK_a in protein environments, which we will apply to protein samples in future studies. These results also suggest the utility of S Kβ and K-edge XAS in time-resolved studies of cysteine dependent enzymes where active site Cys residues undergo changes in ionization and bonding throughout the catalytic cycle, providing an element specific window into the critical S chemistry in these proteins."

Additional Questions:
Urgency: Moderate

Significance: Moderate

Novelty: High

Scholarly Presentation: High

Is the paper likely to interest a substantial number of physical chemists, not just specialists working in the authors' area of research?: Yes
